# Inhibitory Effects of Twenty-Nine Compounds From *Potentilla longifolia* on Lipid Accumulation and Their Mechanisms in 3T3-L1 Cells

**DOI:** 10.3389/fphar.2020.555715

**Published:** 2020-11-09

**Authors:** Qianqian Ma, Li Ye, Wei Li, Shengxi Lin, Xiaoyan Zhao, Chenghua Jin, Guancheng Liu, Huan Liu, Yunpeng Sun, Haidan Yuan, Guangchun Piao

**Affiliations:** ^1^ College of Pharmacy, Yanbian University, Yanji, China; ^2^ Key Laboratory of Natural Resources of Changbai Mountain & Functional Molecules, Yanbian University, Ministry of Education, Yanji, China

**Keywords:** *Potentilla longifolia*, lipid accumulation, SREBP1c, PPARγ, new compound (ganyearmcaooside), 3,8-dimethoxy-5,7,4′- trihydroxyflavone

## Abstract

*Potentilla longifolia* Willd. ex D.F.K.Schltdl., which is a kind of traditional Chinese herb, is often referred to as “Ganyancao” in China, which means “the herb is effective in the treatment of liver inflammation”. Three new (ganyearmcaoosides A and B and ganyearmcaoic acid A; **1**–**3**) and 26 known compounds (**4**–**29**) were isolated from the 95% ethanol extract of the dried aerial parts of this plant, of which 21 were isolated for the first time from this plant. The chemical structures of these compounds were elucidated using NMR and HR-ESI-MS analysis. The inhibitory effects of the 29 compounds with safe concentrations on the lipid accumulation in 3T3-L1 cells were evaluated using photographic and quantitative assessments of lipid contents by Oil Red O staining, and measurement of the triglyceride levels. Comprehensive analysis showed that compound **12** (3,8-dimethoxy-5,7,4′- trihydroxyflavone) showed the best inhibitory effect on lipid accumulation such as reducing the accumulation of oil droplets and triglyceride level, and was superior to the reference in positive control. Western blot analysis and RT-PCR results showed that compound **12** enhanced the phosphorylations of AMPK and ACC, and inhibited the expressions of adipogenesis-related proteins or genes including SREBP1c, FAS, SCD1, GPAT, PPARγ and C/EBPα, and thereby significantly inhibited lipid accumulation in a concentration-dependent manner. *P. longifolia* and its bioactive compounds could be promising as potential therapeutic agents for diseases related to lipid accumulation in the future.

## Introduction

Excessive accumulation of lipids in the human body may lead to many problems, including hypertension, obesity, fatty liver disease, hyperlipidemia, and diabetes. These problems not only disturb the physical and mental health of human beings, but also seriously delay the development of human society and economy (Wada et al., 2007; Ideta et al., 2015; Ma et al., 2018). Solutions to these health problems are often focused on the prevention or suppression of fat accumulation. Human beings have been painstakingly trying to find non-toxic and effective lipid-lowering drugs, including drugs developed from traditional medicine sources. Natural products have been used in traditional medicines since ancient times. Some traditional medicines have been developed and standardized, and continue to contribute to human health. For example, many clinically used drugs of plant origin were derived from traditional medicines (Alves and Rosa, 2007; Li-Weber, 2009; Yuan et al., 2016).

“Chaoyao medicine” is a part of traditional Chinese medicine, mainly used by the people of Chaoxianzu nationality (a minority in China). Chaoyao medicine has formed its own particular details and styles in terms of the methods of use and species of medicinal materials after long-term use and development. A large amount of clinical trial data has been obtained from the long-term practice of Chaoyao medicine. Based on these clinical data, many aspects of Chaoyao medicine including the species and methods of use, have been continuously improved, and thus ensuring its curative effects. The aerial parts, or the whole plant, of *Potentilla longifolia* Wild. ex D.F.K.Schltdl., which is a kind of Chaoyao medicine, is usually used to treat jaundice and other liver injury diseases. Because of its remarkable curative effect in treating hepatitis and other liver inflammation, it is often referred to as “*Ganyearmcao*”, or “*Ganyancao*” in China, which means “the herb is effective in the treatment of liver inflammation” (Piao et al., 2012). Potentilla species have been used for a long time as traditional herbs. This genus has been known and studied since ancient times for its possible therapeutic properties. In in vivo and vitro biological and pharmacological studies of Potentilla species are focused on antiviral, antimicrobial, antiinflammatory, hepatoprotective and antioxidative activities, etc. (Tomczyk and Latte, 2009). For example, one study demonstrated that asiatic acid from *P. chinensis* significantly ameliorated non-alcoholic fatty liver disease by inhibiting the Endoplasmic reticulum stress (ERS) pathway (Wang et al., 2018). The antioxidant experiments showed that the water extracts of both Potentilla species (*P. argentea* and *P. recta*) were the potent 2,2′-azino-bis(3-ethylbenzothiazoline-6-sulphonic acid)[ABTS] scavenger. The methanol extracts of these two Potentilla species were active inhibitors of *α*-glucosidase (Sut et al., 2019). Other study also showed that methanol and water extracts of two Potentilla species (*P. speciosa* L. and *P. reptans* Willd.), and biflavanols and quercetin-3-*O*-*α*- L-rhamnopyranoside-2*''*-gallate isolated from *P. anserina* L. displayed good antioxidant activity (Uysal et al., 2017; Yang et al., 2020). Several tannins and flavonoids have been reported to be present in *P. longifolia* which was also known as *P. viscosa*. A few pharmaceutical research such as anti-oxidant activity of *P. longifolia* was also conducted (Wollenweber and Dorr, 2008; Tomczyk and Latte, 2009). Considering the traditional effect of *P. longifolia* on hepatitis, and the close relationship among hepatitis, fatty liver and lipid accumulation, we conducted the experiment of compounds isolated from *P. longifolia* on inhibitory effects on lipid accumulation in our previous study, and obtained good results. Therefore, the inhibitory effects of the herb on lipid accumulation was further studied.

AMP-activated protein kinase (AMPK) regulates fat and carbohydrates, adjusts the energy balance in cells, and is closely related to elements of lipid metabolism, such as adipogenesis and lipogenesis; thus, AMPK affects lipid accumulation in conditions such as obesity (Ha et al., 2016; Ma et al., 2018). After activation, AMPK inhibits the expression of Sterol regulatory element-binding protein 1c (SREBP1c), CCAAT/enhancer-binding protein *α* (C/EBPα), and Peroxisome proliferators-activated receptor-gamma (PPARγ) and their downstream genes, thereby inhibiting adipogenesis and lipogenesis, and regulating lipid metabolism (Gan et al., 2017). In addition, AMPK can inhibit lipid synthesis by inhibiting glycerol-3-phosphate acyltransferase (GPAT), the first key enzyme involved in catalyzing triglyceride (TG) synthesis, which results in lipid regulation (Lindén et al., 2004; Henriksen et al., 2013). SREBP1c is a transcription factor involved in adipogenesis; SREBP1c activates genes and enzymes, including fatty acid synthase (FAS) and Stearoyl-Coenzyme A desaturase 1 (SCD1). PPARγ and C/EBPα are considered to be the main regulators of adipogenesis. PPARγ increases the expression of C/EBPα, which activates many adipocyte-specific genes, including SCD1. SCD1 is closely related to adipocyte maturation and the levels of TGs and fatty acids, which preferentially regulate adipogenesis (Ha et al., 2016; Ma et al., 2018). In addition, PPARγ combined with C/EBPα increased the expression of adipocyte-related genes and induced the formation of mature adipocytes in the late stage of adipogenesis (Moseti et al., 2016).

In a herbal medicine, there must be active chemicals acting alone or in concert to achieve the desired effects. Therefore, it is very important to study the chemical constituents in medicinal plants (Yuan et al., 2016). In this study, the chemical constituents of *P. longifolia* and their anti-lipid accumulation activity and mechanisms of action were investigated.

## Materials and Methods

### General Experimental Procedures for Separation and Identification of Chemical Structures of Compounds

The NMR spectra were recorded using Bruker AV500/300MHz spectrometer (Bruker, Fallanden, Switzerland). High-resolution electrospray ionisation mass spectra (HR-ESI-MS) were obtained using a Bruker microTOF QII mass spectrometer (Bruker Daltonics, Fremont, CA, United States). Optical rotation was measured using a Rudolph Autopol I automatic polarimeter (Rudolph Research Analytical, Hackettstown, NJ, United States). Column chromatography was performed using silica gel (200-300 mesh, Qingdao Haiyang Chemical Co., Ltd, Qingdao, China). Sephadex LH-20 was purchased from GE Healthcare (United States). TLC was performed using precoated silica gel 60 RP-18 F254s glass and aluminum plates (200 mm × 200 mm, Merck, Germany).

### Chemicals and Reagents

The 3T3-L1 cells were purchased from ATCC (Manassas, VA, United States). Dulbecco’s modified Eagle’s medium (DMEM), fetal calf serum (FCS), fetal bovine serum (FBS), and penicillin−streptomycin were purchased from Gibco by Life Technologies (Grand Island, NY, United States). The TG assay kit was purchased from Nanjing Jiancheng Bioengineering Institute, China. Protein extraction, EASY BLUE total RNA extraction, and ECL-reagent kits were from Intron Biotechnology Inc. (Beverly, MA, United States). The Bio-Rad protein assay kit was from BioRad Laboratories (Hercules, CA, United States). ODS (50 μm) was obtained from YMC (Japan). All other chemicals and solvents were analytical grade.

### Plant Material

The aerial parts of *Potentilla longifolia* Wild. Ex Schlecht. was acquired in Changbai Mountain, Jilin Province, China, in October 2014. The sample was authenticated by Prof. HZ Lv of College of Pharmacy, Yanbian University (voucher specimen: ID-2014106, stored in Chaoyao Herbarium of Yanbian University).

### Extraction and Isolation

The aerial parts of *Potentilla longifolia* (5 kg) were extracted by refluxing in 20 L 95% ethanol (EtOH)/H_2_O (4 h × 3). The resulting extract (1030 g) was dispersed in H_2_O (5 L) and extracted subsequentially with petroleum ether, Ethyl acetate (EtOAc), and n-butyl alcohol (n-BuOH) (3 × 5 L, respectively), yielding petroleum ether (164.5 g), EtOAc (254.2 g), n-BuOH (203.9 g), and water (344.5 g) fractions.

The EtOAc fraction (254.2 g) was subjected to silica gel column chromatography with a gradient of petroleum ether-EtOAc (100:1, 10:1, 5:1, 2:1, 1:1, 0:100) to give six subfractions (Fr. E-1^**__**^6). Fr. E-2(7.32 g) was separated using a silica gel column (petroleum ether-EtOAc, 50:1–1:1) to give 8 subfractions (Fr. E-2-1^**__**^8). Fr. E-2-8 was re-chromatographed on a silica gel column repeatedly to obtain compound **29** (22 mg) and **9** (8 mg). Fr. E-3 (40.96 g) was fractionated into 16 subfractions (Fr. E-3-1^**__**^16) using a a silica gel chromatography with a gradient of petroleum ether-EtOAc (25:1, 1:1). E-3-9 was recrystallized to obtain compound **7** (10 mg) and E-3-12 was recrystallized to obtain compound **26** (130 mg). Fr. E-3-7 (4.36 g) was subjected to silica gel column chromatography with a gradient of CH_2_Cl_2_-methanol (MeOH)(30:1-5:1) to give 5 subfractions (Fr. E-3-7-1^**__**^5). Fr. E-3-7-5 (1.76 g) was separated using a silica gel column to obtain compound **27** (30 mg). Fr. E-4 was fractionated into 6 subfractions (Fr. E-4-1^**__**^6) using a a silica gel chromatography with a gradient of CH_2_Cl_2_-MeOH (500:1-10:1). Fr. E-4-3 was recrystallized to obtain compound **11** (56 mg). Fr. E-4-2 was separated using a silica gel column with a gradient of CH_2_Cl_2_-MeOH repeatedly to obtain compound **10** (6 mg). Fr. E-4-3 was subjected to silica gel column chromatography with a gradient of CH_2_Cl_2_-MeOH and reverse-column (ODS-A) with a gradient of MeOH-H_2_O to yield compounds **21** (12 mg), **8** (11 mg) and **22** (16 mg). Fr. E-6(101.5 g) was fractionated into 8 subfractions (Fr. E-6-1^__^8) using a a silica gel chromatography with a gradient of CH_2_Cl_2_-MeOH (90:1-1:1). Fr. E-6-3 (5.4 g) was subjected to silica gel column chromatography with a gradient of CH_2_Cl_2_-MeOH and one of the obtained subfraction (Fr. E-6-3-1) was separated using a Sephadex LH-20 column to obtain compound **12** (20 mg). Fr. E-6-4 (13.1 g) was fractionated into 7 subfractions (Fr. E-6-4-1^__^7) using a a silica gel chromatography with a gradient of petroleum ether-EtOAc. Fr. E-6-4-6, Fr. E-6-4-1, and Fr. E-6-4-2 were subjected respectively to silica gel column chromatography with a gradient of CH_2_Cl_2_-MeOH or petroleum ether-EtOAc to yield compounds **19** (30 mg), **28** (65 mg), **13** (15 mg) and **3** (35 mg). Fr. E-6-6 (23.8 g) was subjected to silica gel column chromatography with a gradient of CH_2_Cl_2_-MeOH and then a Sephadex LH-20 column repeatedly to yield compounds **18** (16 mg), **14** (43 mg), **6** (5 mg) and **1** (3 mg).

The n-BuOH (203.9 g) fraction was subjected to macroporous resin column chromatography with a gradient of EtOH-H_2_O (H_2_O, 25% EtOH, 50% EtOH, 75% EtOH, 95% EtOH) to give six subfractions (Fr. B-1^**__**^5). Fr. B-2 was separated using reverse-column(ODS-A) with a gradient of MeOH-H_2_O repeatedly to obtain compound **15** (18 mg). Fr. B-3 was subjected to silica gel column chromatography with a gradient of CH_2_Cl_2_-MeOH-H_2_O to afford 15 subfractions (Fr. B-3-1^__^15). Fr. B-3-3(1.701g) was separated using reverse-column(ODS-A), silica gel column and Sephadex LH-20 column repeatedly to yield compounds **5** (3.9 mg), **25** (2.1 mg), **23** (3.1 mg) and **4** (2.5 mg). Fr. B-3-4 (1.2 g) was subjected to silica gel column chromatography, Sephadex LH-20 column chromatography and reverse-column(ODS-A) chromatography repeatedly to afford compounds **24** (3.6 mg), **20** (8.2 mg), **17** (9.1 mg) and **2** (19.4 mg). Fr. B-3-5 (556 mg) was separated using silica gel column with a gradient of petroleum ether-EtOAc (20:1, 15:1, 10:1,5:1) to obtain compound **16** (8.2 mg).

Compound **1**: white, amorphous powder; [α]25D = −44.43(c = 0.05, CH_3_OH); HR-ESI-MS *m*/*z* 387.1643 [M + H]^+^ (calcd for C_18_H_27_O_9_, 387.1650); ^1^H NMR (CD_3_OD, 300 MHz) and ^13^C NMR data (CD_3_OD, 75 MHz), see Table 1.

**TABLE 1:** ^1^H NMR and ^13^C NMR data of compound **1** in CD_3_OD (300 MHz) and compound **2** in DMSO (500 MHz).

compound	1		2	
Position	*δ*_H_ (*J* in Hz)	δ_C_	*δ*_H_ (*J* in Hz)	δ_C_
1	—	114.2	—	114.7
2	—	157.8	—	155.0
3	6.27, d (1.9)	96.9	6.43, d (1.8)	95.4
4	—	162.4	—	159.2
5	6.13, d (1.9)	94.7	6.36, d (1.8)	94.3
6	—	160.0	—	156.9
1′	—	207.6	—	202.7
2′	2.64, m	55.0	2.61, qd (16.8, 6.8)	53.2
3′	2.08, m	26.3	2.05, dp (13.4, 6.8)	24.0
4′	0.89, d (6.6)	23.1	0.88, d (6.7)	22.5
5′	0.89, d (6.6)	22.9	0.88, d (6.7)	22.3
1′′	4.79, d (7.2)	103.0	4.85, d (7.7)	100.4
2′′	3.30, m	74.9	3.15, m	73.3
3′′	3.30, m	78.3	3.42, m	77.1
4′′	3.30, m	71.2	3.09, m	70.0
5′′	3.30, m	78.0	3.26, m	76.8
6′′	3.85, dd (12.0, 1.9) 3.65, dd (12.0, 5.2)	62.5	3.72, dd (10.4, 5.8) 3.42, m	60.9
1′′′	—	—	4.89, d (7.3)	99.8
2′′′	—	—	3.26, m	73.2
3′′′	—	—	3.42, m	77.1
4′′′	—	—	3.09, m	69.8
5′′′	—	—	3.26, m	76.8
6′′′	—	—	3.72, dd (10.4, 5.8) 3.42, m	60.9
6-OCH_3_	3.71, s	56.1	3.69, s	55.8

Compound **2**: white, amorphous powder; [α]25D = −94.95 (c = 0.21,CH_3_OH); HR-ESI-MS *m*/*z* 549.2181 [M + H]^+^ (calcd for C_24_H_37_O_14_, 549.2178); ^1^H NMR (DMSO, 500MHz) and ^13^C NMR data (DMSO, 125 MHz), see Table 1.

Compound **3**: white, amorphous powder; HR-ESI-MS *m*/*z* 487.3409 (calcd for C_30_H_47_O_5_, 487.3418); [α]25D = 85.57(c = 0.20,CH_3_OH); ^1^H NMR (CD_3_OD, 300MHz) and ^13^C NMR data (CD_3_OD, 75 MHz), see Table 2.

**TABLE 2:** ^1^H NMR and ^13^C NMR data of compound **3** in CD_3_OD (300 MHz).

compound	3	
Position	*δ*_H_ (*J* in Hz)	δ_C_
1	—	215.3
2	3.07, t (12.0) 2.23, dd (12.0, 4.6)	45.1
3	3.33, m	79.6
4	—	40.4
5	0.83, overlapped	55.7
6	1.61, m	19.0
7	1.51, m 1.29, m	34.1
8	—	40.9
9	2.30, m	40.1
10	—	53.8
11	2.33, m 1.78, m	26.5
12	5.24, br s	130.0
13	—	139.3
14	—	42.8
15	1.76, m	29.6
16	2.52, m 1.49, m	26.6
17	—	49.1
18	2.45, m	55.2
19	—	73.5
20	1.26, m	43.1
21	1.67, m	27.3
22	1.66, m	38.9
23	0.97, s	16.6
24	1.00, s	29.0
25	1.28, s	15.4
26	0.82, s	18.1
27	1.31, s	24.8
28	—	182.3
29	1.18, s	27.0
30	0.90, d (6.6)	16.6

Other spectra of the three new compounds, including ^1^H−^1^H COSY, HMBC, HMQC, NOESY, HR-ESI-MS, etc., see Supplementary data (Supplementary Figures S1–S17).

### Cell Culture and Cytotoxicity Assay

The 3T3-L1 cells were cultured in DMEM containing 10% FCS, 100 unit/ml penicillin and 100 μg/ml streptomycin at 37°C in an atmosphere of 5% CO_2_. For the cytotoxicity assay, 3 × 10^4^ 3T3-L1 cells per well were cultured in 96-well plates and treated with the thirty compounds at the concentrations of 0, 10, 20, 40, or 80 μM for 96 h, respectively. Three parallel wells were set at per concentration. The cytotoxicities of these compounds were determined by the MTT assay. Absorbance was measured at 540 nm to determine viable cell numbers in wells.

The 3T3-L1 cells (5 × 10^5^ cells per well) were cultured in 6-well culture plates. The 3T3-L1 cells were divided into a normal control (CON) group, a differentiated control treated with differentiation medium (DM) group, a differentiated positive control treated with DM plus pioglitazone (PIO) group, 29 compound treatment groups treated with the each of 29 compounds (in which compound 26, ursolic acid, was used as a reference compound in positive control), respectively. The 29 compound treatment groups were treated with 40 μM of compounds **1**, **2**, **4**, **5**, **8**, **10**, **11**, **12**, **13**, **14**, **16**, **17**, **18**, **20**, **21**, **22**, **23**, **24**, **27**, **29**, 20 μM of compounds **3**, **6**, **7**, **9**, **15**, **19**, **26**, **28**, and 10 μM of compound **25**, respectively.

When the cells were incubated until confluence (day 0), they were exposed to DM I (DMEM, 5% FBS, 10 μg/ml insulin, 1 mM dexamethasone, and 0.5 mM 3-isobutyl-1-methylxanthine) for 4 days (day 4); then, except for the CON group, the cells were exposed to DM II (DMEM containing 5% FBS and 10 μg/ml insulin) for two more days (day 6); and then, except for the CON group, the cells were exposed to DM III (DMEM containing 5% FBS) for two more days (day 8). From day 0 to day 4 during adipocyte differentiation, the cells in thirty compound treatment groups were treated with corresponding compounds with corresponding concentrations.

### Oil-Red O Staining

After discarding the medium in the 6-well plate, the differentiated cells were washed twice with phophate-buffered saline (PBS) and fixed with 10% formalin for 1 h. The cells then were stained with 1 ml Oil-Red O solution for 2 h to observe the differentiation of 3T3-L1 cells. The pictures were taken by an Olympus microscope. After that, 6-well plates were treated with isopropanol, and the absorbances were determined at 540 nm to evaluate the lipid accumulation.

### Measurement of the TG Level

The 3T3-L1 cells were lysed in lysis buffer which included 25 mM sucrose, 20 mM Tris−HCl, 1 mM EGTA, and 1 mM EDTA, and the cells were collected and centrifugated at 13,000 rpm for 15 min. A TG assay kit was used to measured the levels of TG in accordance with the instructions of the manufacturer. A Bio-Rad protein assay reagent (Bio-Rad Laboratories) was used to determine the concentration of protein in accordance with the manufacturer’s instructions.

Furthermore, the experimental details in the Western blot analysis, RNA isolation and reverse transcription polymerase chain reaction (RT-PCR) and statistical analysis, including primers and sequences, etc., followed the relevant contents of our previous paper (Ma et al., 2018).

## Results and Discussion

### Identification of Compounds 1–29

#### Compound 1

Compound **1** was obtained as a white amorphous powder, the molecular formula was assigned as C_18_H_26_O_9_ based on the [M + H]^+^ peak at *m*/*z* 387.1643 (calcd for C_18_H_27_O_9_, 387.1650) in the HR-ESI-MS data (Supplementary Figure S5). The observed NMR data suggest that compound **1** is a phloroglucinol glucoside which was similar to 2-*β*-D-gluco-pyranosyloxy-4-methoxy- 6-hydroxyisovalero- phenone) (Wang et al., 2014a), except the positions of methoxy group and hydroxyl group were exchanged.

The ^1^H NMR spectrum of **1** (Table 1) showed one tetra-substituted phenyl moiety signals at *δ*_H_ 6.27 (1H, d, *J* = 1.9 Hz, H-3), 6.13 (1H, d, *J* = 1.9 Hz, H-5), an anomeric proton at *δ*_H_ 4.79 (1H, d, *J* = 7.2 Hz, H-1′′), along with six oxygenated protons (*δ*_H_ 3.85, 3.65, 3.27-3.40), suggesting the presence of a *β*-glucopyranosyl moiety. Other characteristic proton signals indicated one methoxy proton at *δ*_H_ 3.71 (3H, s, 6-OC*H*_3_), one methylene proton at *δ*_H_ 2.64 (2H, m, H-2′), a multiplet methine at *δ*_H_ 2.08 (1H, m, H-3′), and two methyl signals at *δ*_H_ 0.89 (6H, d, *J* = 6.6 Hz, H-4′, H-5′).

**FIGURE 1:**
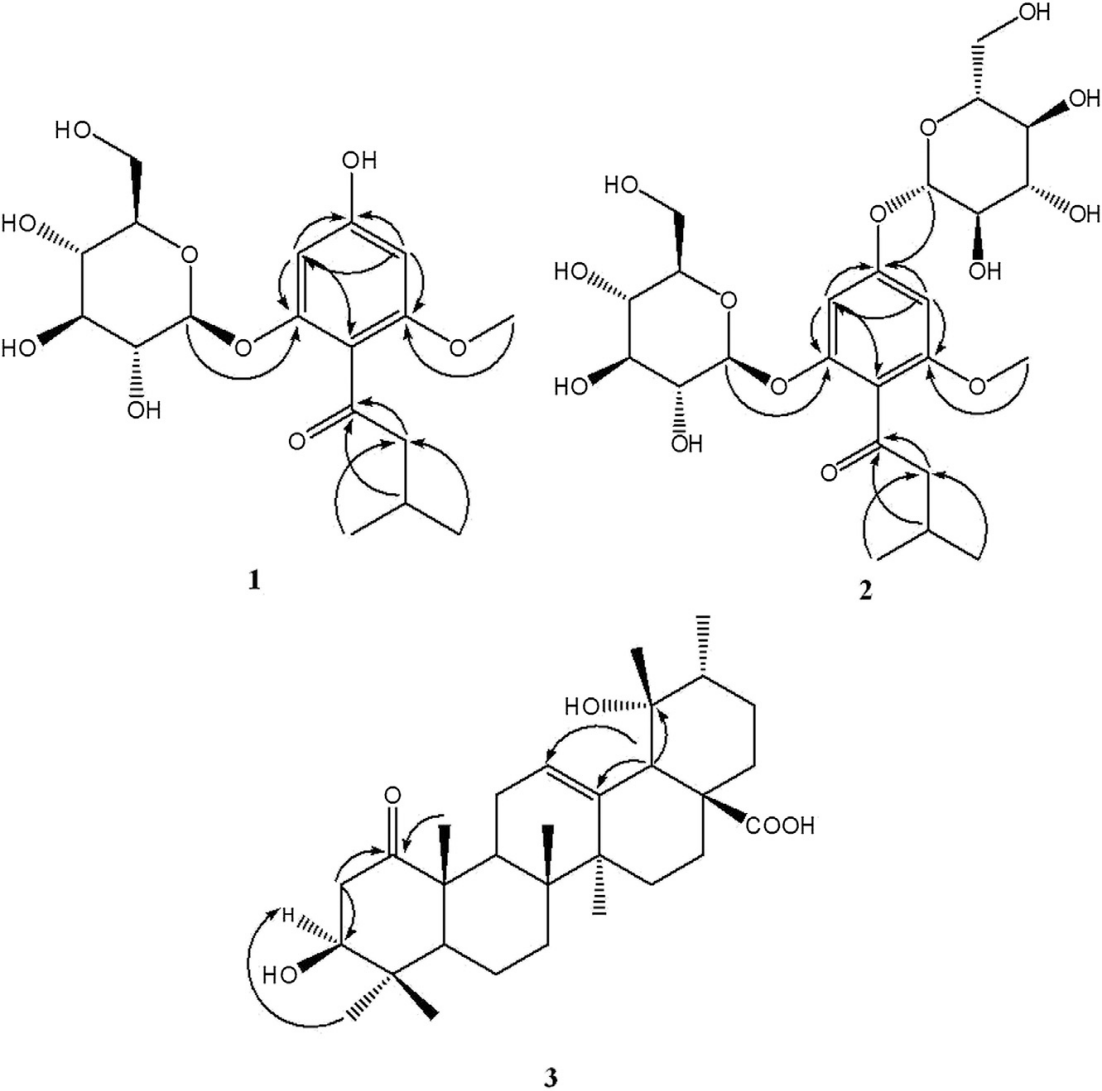
Key HMBC (H→C) correlations of compounds **1**-**3**.

**FIGURE 2:**
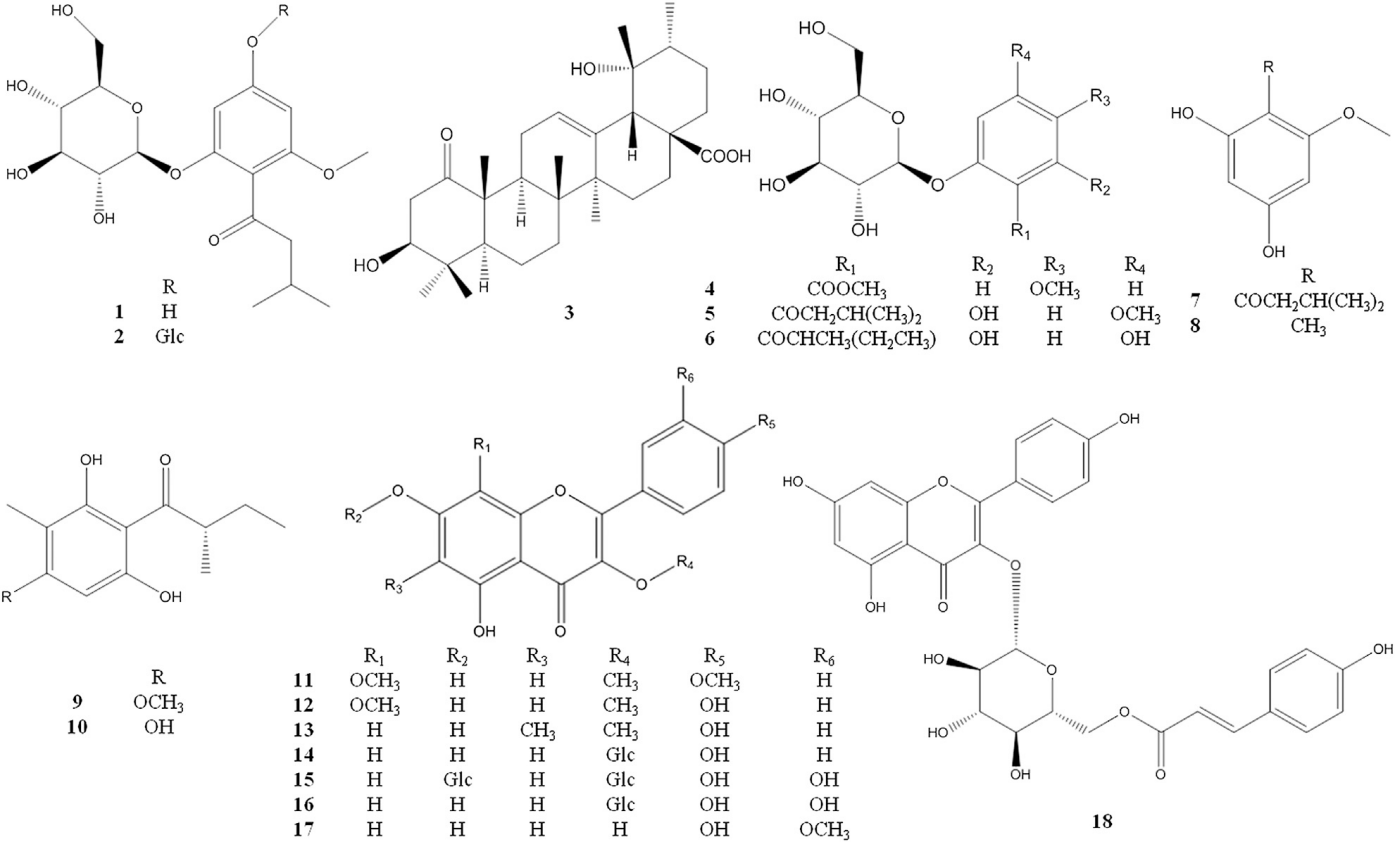
The chemical structures of compounds **1**–**18** isolated from *Potentilla longifolia.*

The ^13^C NMR spectrum displayed 18 carbon signals including a carbonyl carbon at *δ*_C_ 207.6 (C-1′), one benzene ring bearing six aromatic carbons (*δ*_C_ 162.4, 160.0, 157.8, 114.2, 96.9, 94.7), a glucopyranosyl moiety containing six signals (*δ*_C_ 103.0, 78.3, 78.0, 74.9, 71.2, 62.5), one methoxy carbon at *δ*_C_ 56.1 (6-O*C*H_3_), a methylene signal at *δ*_C_ 55.0 (C-2′), one methine carbon at *δ*_C_ 26.3 (C-3′), and two methyls (*δ*_C_ 23.1 and 22.9). All the above NMR data of **1** exhibited the skeleton of this compound belonged to the phenol glycosides. The HMBC correlation between *δ*_H_ 3.71 (OC*H*_3_) and *δ*_C_ 160.0 (C-6) indicated that the methoxy group was connected to C-6. The sugar moiety was identified as D-glucose based on the acid-hydrolyzed by GC analysis and the glycosidic linkage was confirmed on the C-2 position by the HMBC correlation between *δ*_H_ 4.79 (H-1′′) and *δ*_C_ 157.8 (C-2) (Figure 1). Thus, the structure of compound **1** was established as 2-*β*-D-gluco-pyranosyloxy-6-methoxy-4- hydroxyiso- valerophenone (named as ganyearmcaooside A). The ^1^H (300 MHz) and ^13^C NMR data for compound **1** were presented in Table 1 while its structure was shown in Figure 2.

#### Compound 2

Compound **2** was obtained as a white amorphous powder, the molecular formula was assigned as C_24_H_36_O_14_ based on the [M + H]^+^ peak at *m*/*z* 549.2181 (calcd for C_24_H_37_O_14_, 549.2178) in the HR-ESI-MS data (Supplementary Figure S10). The ^1^H and ^13^C NMR data revealed that the framework of **2** was the same as **1**, with an additional glucopyranosyl moiety at C-4 position, as shown in Figure 2.

The ^1^H NMR spectrum of compound **2** (Table 1) showed two aromatic protons at *δ*_H_ 6.43 (1H, d, *J* = 1.8 Hz, H-3), 6.36 (1H, d, *J* = 1.8 Hz, H-5), two anomeric protons at *δ*_H_ 4.89 (1H, d, *J* = 7.3 Hz, H-1′′′), 4.85 (1H, d, *J* = 7.7 Hz, H-1′′), along with twelve oxygenated protons (*δ*_H_ 3.72-3.09), indicating the presence of two *β*-glucopyranosyl moieties, one methoxy proton at *δ*_H_ 3.69 (3H, s, 6-OC*H*_3_), one methylene group at *δ*_H_ 2.61 (2H, qd, *J* = 16.8, 6.8 Hz, H-2′), a methine proton at *δ*_H_ 2.05 (1H, dp, *J* = 13.4, 6.8 Hz, H-3′), and two methyls at *δ*_H_ 0.88 (6H, d, *J* = 6.7 Hz, H-4′, H-5′). The ^13^C NMR spectrum displayed 24 carbon signals including a carbonyl carbon at *δ*_C_ 202.7 (C-1′), one benzene ring bearing six aromatic carbons (*δ*_C_ 159.2, 156.9, 155.0, 114.7, 95.4, 94.3), two glucopyranosyl moieties containing twelve carbon signals, one methoxy carbon at *δ*_C_ 55.8 (6-O*C*H_3_), a methylene signal at *δ*_C_ 53.2 (C-2′), one methine carbon at *δ*_C_ 24.0 (C-3′), and two methyls (*δ*_C_ 22.5 and 22.3). All the observed NMR data were similar to those of compound **1**, except that the hydroxy group at C-4 position was replaced by a sugar moiety in compound 2, and the sugar moiety was also identified as D-glucose by the same acid-hydrolyzed method. This sugar moiety location was further supported by the HMBC correlation between *δ*_H_ 4.89 (H-1′′′) and *δ*_C_ 159.2 (C-4) (Figure 1). Thus, compound **2** was elucidated as 2-*β*-D-glucopyranosyloxy-6-methoxy-4-*β*-D- glucopyranosyloxy- isovalerophenone (named as ganyearmcaooside B). The ^1^H (500 MHz) and ^13^C NMR data for compound **2** were presented in Table 1 while its structure was shown in Figure 2.

#### Compound 3

Compound **3** was isolated as a white amorphous powder, the molecular formula was established as C_30_H_46_O_5_ by [M+H]^+^ peak at *m*/*z* 487.3409 (calcd for C_30_H_47_O_5_, 487.3418) in the HR-ESI-MS spectrum (Supplementary Figure S17). Most of the peaks in the ^1^H and ^13^C NMR spectra of this compound match those of pomolic acid (Mei et al., 2016) with an additional carbonyl group at position C-1, as shown in Figure 2.

The ^1^H NMR spectrum (Table 1) contained an olefinic proton at *δ*_H_ 5.24 (1H, br s, H-12), an oxygenated methine proton at *δ*_H_ 3.33 (1H, m, H-3), six tertiary methyls at *δ*_H_ 1.31, 1.28, 1.18, 1.00, 0.97, 0.82 (each 3H, s), and one secondary methyl proton at *δ*_H_ 0.90 (3H, d, *J* = 6.6 Hz, H-30).

The ^13^C NMR spectrum (Table 2) revealed 30 carbon signals, which indicated that the framework of compound **3** is a ursane-type triterpene, the carbon C-1 at *δ*_C_ 215.3 is a carbonyl carbon while C-28 at *δ*_C_ 182.3 corresponds to a carboxylic carbon, the other characteristic carbon signals including a pair of olefinic carbon signals at *δ*_C_ 139.3 (C-13) and 130.0 (C-12), a hydroxymethine at *δ*_C_ 79.6 (C-3), an oxygenated quaternary carbon at *δ*_C_ 73.5 (C-19), and seven methyls at *δ*_C_ 29.0 (C-24), 27.0 (C-29), 24.8 (C-27), 19.0 (C-26), 16.6 (C-23, C-30), and 15.4 (C-25). The carbons C-2 and C-10 were downfield resonating at *δ*_C_ 45.1 and at *δ*_C_ 53.8 also indicating the carbonyl group located at C-1 position. In the HMBC spectrum (Figure 1), the methylene signal at H-2 (*δ*_H_ 3.07, 2.33) correlated with C-1 (*δ*_C_ 215.3), the methyl proton at H-25 (*δ*_H_ 1.28) correlated with C-1 (*δ*_C_ 215.3), suggesting that the carbonyl group should be located at C-1. The relative configuration of compound **3** was determined by the NOESY experiment, the key correlations between H-3 (*δ*_H_ 3.33) and H-23 (*δ*_H_ 0.97), H-18 (*δ*_H_ 2.45) and H-29 (*δ*_H_ 1.18), showing that the 3-hydroxyl group and 19-hydroxyl group were assigned as *β*-equatorial and *α*-equatorial, respectively. Consequently, the structure of compound **3** was determined to be 3*β*, 19*α*- dihydroxy-1-oxours- 12-en-28-oic acid (named as ganyearmcaoic acid A). The ^1^H (300 MHz) and ^13^C NMR data for compound **3** were presented in Table 2 while its structure was shown in Figure 2.

Other spectra of the three new compounds, including ^1^H−^1^H COSY, HMBC, HMQC, NOESY, HR-ESI-MS, etc., see Supplementary data (Supplementary Figures S1-S17).

#### Compounds 4–29

**FIGURE 3:**
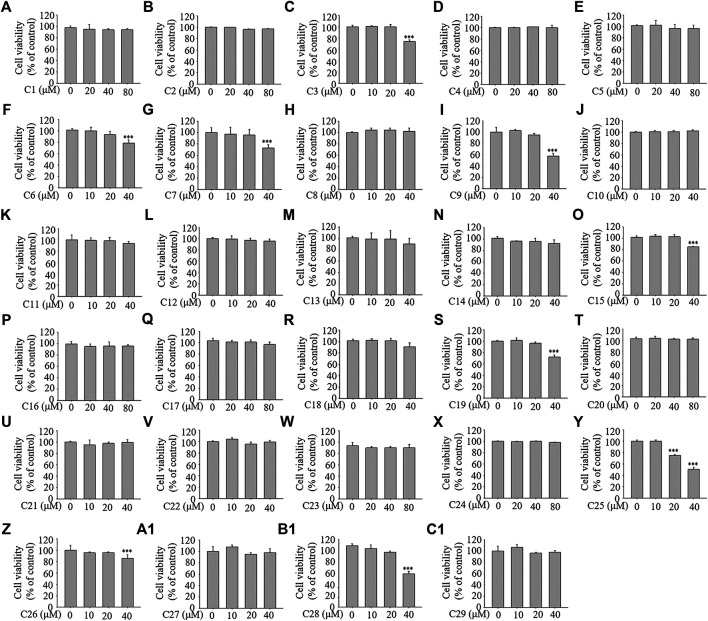
The chemical structures of compounds **19**–**29** isolated from *Potentilla longifolia.*

The known compounds **4**–**29** were identified as 2-O-β-glucopyranosyl -5- methoxy- benzoic acid methyl ester (**4**) (Kurimoto et al., 2011), 2-β-D- glucopyranosyloxy-4-methoxy-6- hydroxy- isovalero-phenone (**5**) (Wang et al., 2014a), 1-[(2-methylbutyryl) phloroglucinol]-β-d- glucopyranoside (**6**) (Bohr et al., 2005), 1-(2,4-dihydroxy-6- methoxyphenyl)-3- methyl-1-butanone (**7**) (Wang et al., 2016), 1-O-methyl-2- methylphloroglucinol (**8**) (Arnone et al., 1990), aspidinol D (**9**) (Wang et al., 2010), 2-methyl-1- (2,4,6-trihydroxy- 3-methylphenyl)butan-1-one (**10**) (Wu et al., 2019), 5,7-dihydroxyl-3,8,4′- trimethoxylflavone (**11**) (Liu et al., 2015), 3,8-dimethoxy-5,7,4′-trihydroxyflavone (**12**) (Gedara et al., 2003), 5,7,4′- trihydroxy-3-methoxy-6-methylflavone (**13**) (Rabesa and Voirin, 1978), astragalin (**14**) (Qian et al., 2004), quercetin-3,7-di-O-β-d- glucopyranoside (**15**) (Huang et al., 2002), hirsutrin (**16**) (Shu et al., 2011), isorhamnetin (**17**) (Wang et al., 2014b), trans-tiliroside (**18**) (Itoh et al., 2009), 6-methylapigenin (**19**) (Wasowski et al., 2002), apigenin (**20**) (Miao et al., 2008), farrerol (**21**) (Zhou et al., 2007), 8-demethylfarrerol (**22**) (Li et al., 2014), pinoresinol-4-O-β-D-glucoside (**23**) (Cha et al., 2018), schilignan F (**24**) (Yang et al., 2018), (6R,9R)-3-oxo-α-ionol 9-O-β-glucopyranoside (**25**) (Yang et al., 2010), ursolic acid (**26**) (Jia et al., 2017), *β*-sitosterol (**27**) (Liu and Yang, 2007), 2′, 3′-dihydroxy propylpentadecanoate (**28**) (Wang et al., 2011), octyl decanoate (**29**) (Hosseini-Sarvari and Sodagar, 2013) (see Figures 2 and 3).

Three of these thirty compounds were new compounds (ganyearmcaooside A and B, ganyearmcaoic acid A, **1**-**3**), and other eighteen compounds (**4**–**13**, **15**, **16**–**19**, **21**–**23**, **25**, **29**) were also isolated for the first time from *P. longifolia*.

### The Effects of the 29 Compounds on Cell Viability of 3T3-L1 Cells

**FIGURE 4:**
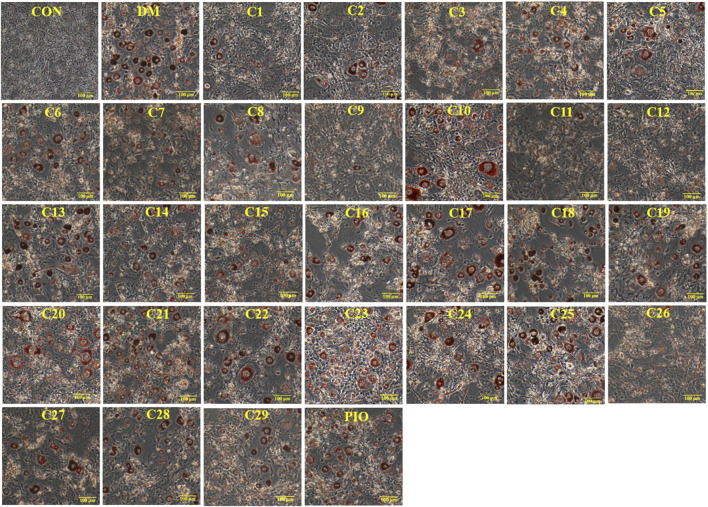
The effects of the 29 compounds on cell viability of 3T3-L1 cells. 3T3-L1 cells were treated with 29 compounds for 96 h at the concentration of 0, 10, 20, 40, 80 μM, respectively. The cytotoxicites of these compounds were determined by the MTT assay. Data represent the mean ± S.D. of three separate experiments. *** *p* < 0.001 as compared with 0 µM group.

To examine the cellular toxicity, 3T3-L1 cells were treated with each of the 29 compounds for 96 h at various concentrations (0–80 μM). The MTT assay showed that concentrations from 0–40 μM of compounds **1**, **2**, **4**, **5**, **8**, **10**, **11**, **12**, **13**, **14**, **16**, **17**, **18**, **20**, **21**, **22**, **23**, **24**, **27**, and **29**; from 0–20 μM of compounds **3**, **6**, **7**, **9**, **15**, **19**, **26** (reference compound in positive control), and **28**; and from 0–10 μM of compound **25** showed no toxicity (see Figure 4). The compounds at the corresponding concentrations were employed in the subsequent experiments.

### Inhibitory Effects of the 29 Compounds on Lipid Accumulation from Oil-Red O Staining in 3T3-L1 Cells

Ursolic acid, which is a pentacyclic triterpene natural compound and can be isolated from the leaves, flowers and fruits of many medicinal herbs such as *Rosmarinus officinalis*, has aroused great interest for its inhibitory effects on lipid accumulation (Chu et al., 2015; Katashima et al., 2017). In this experiment, ursolic acid was isolated as one of the 30 compounds from *P. longifolia* and therefore it was selected as the reference compound in the positive control.

**FIGURE 5:**
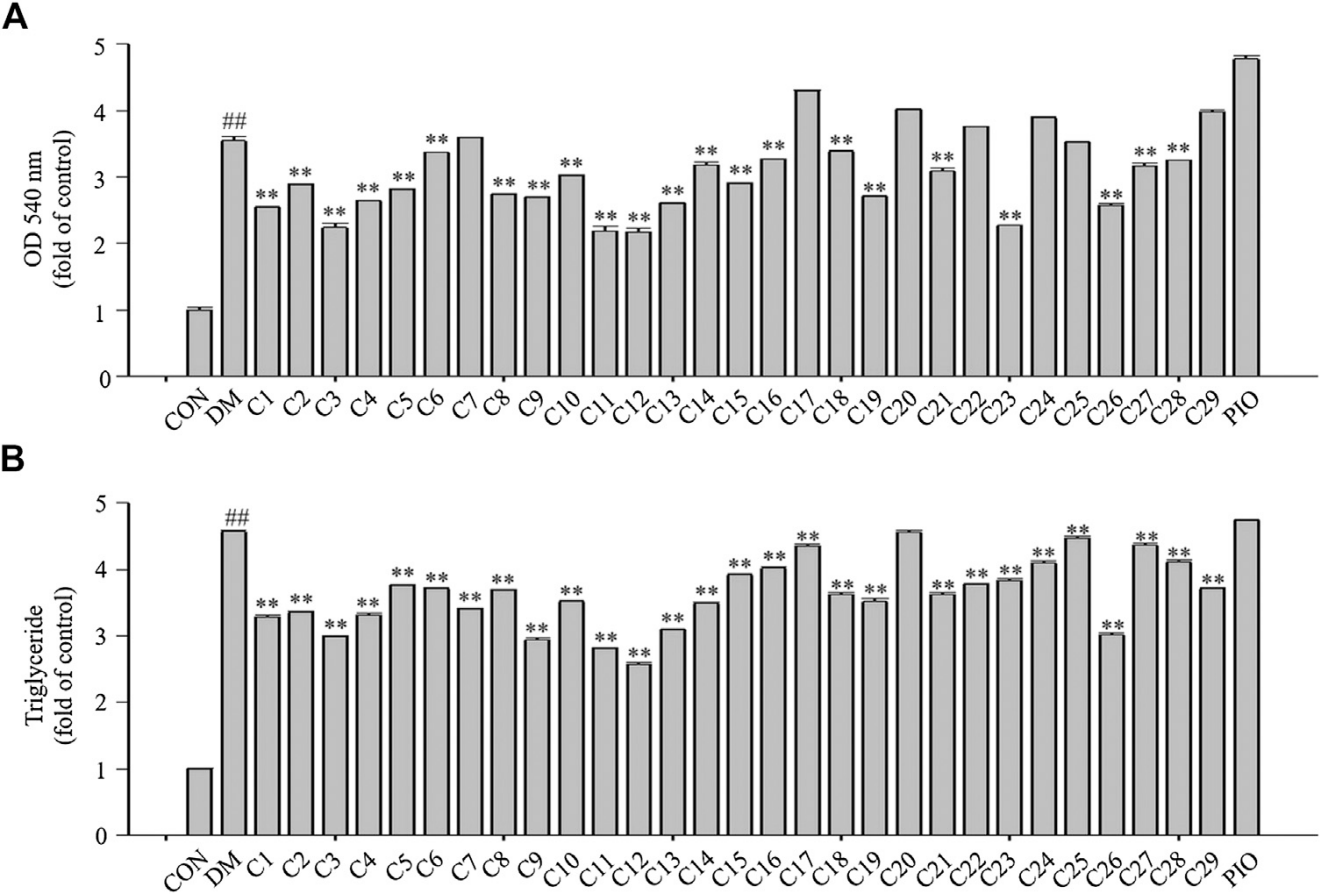
Inhibitory effects of the 29 compounds on lipid accumulation from Oil-Red O staining in 3T3-L1 cells. Lipid accumulation was evaluated by Oil-Red O staining in 3T3-L1cells. 3T3-L1cells were divided into normal control (Con) group, differentiated control treated with differentiation medium (DM) group, differentiated positive control treated with differentiation medium plus pioglitazone (PIO) group, and thirty individual treatment groups treated with thirty compounds alone respectively.

The results of Oil Red O staining showed that compared with the differentiation medium (DM) group, the accumulation of oil droplets in the 3T3-L1 cells treated with compounds **1**, **2**, **3**, **5**, **8**, **9**, **11**, **12**, **13**, **15**, **19**, and **26** (reference compound in the positive control) were decreased and the differentiation of 3T3-L1 cells was inhibited to some extent. Among them, compounds **1**, **9**, **11** and **12** were similar to compound 26 (positive control) in terms of reducing the accumulation of oil droplets. (see Figure 5).

### The Effects of the 29 Compounds on Lipid Accumulation in 3T3-L1 Cells

**FIGURE 6:**
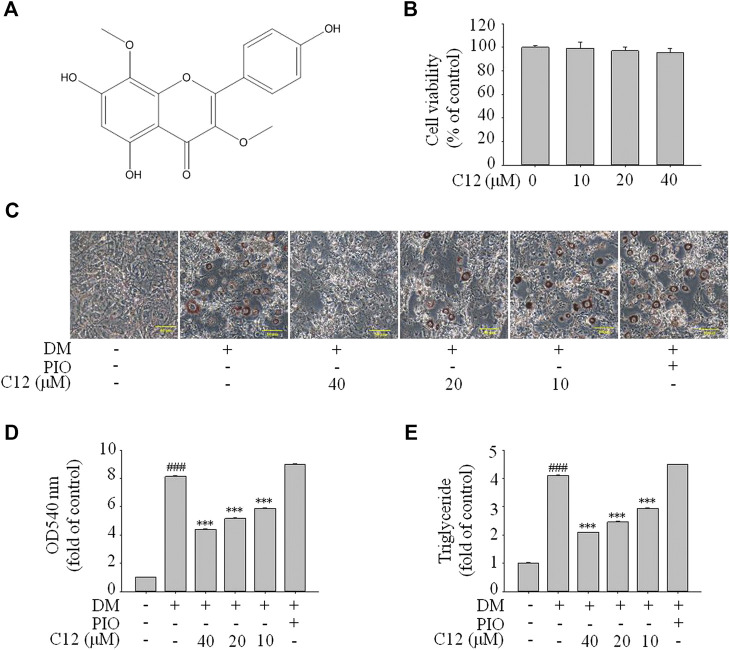
The effects of the 29 compounds on lipid accumulation in 3T3-L1 cells. 3T3-L1 cells were treated with 29 compounds alone respectively. **(A)** After Oil-Red O staining, 3T3-L1 cells were treated with isopropanol and lipid accumulation contents were measured. Data represent the mean ± S.D. of three separate experiments. **(B)** The triglyceride levels were measured by using TG assay Kit. Data represent the mean ± S.D. of three separate experiments. ## *p* < 0.01 as compared with CON group; ** *p* < 0.01compared with DM group. The significant difference was only shown to *p* < 0.01, even when the difference was *p* < 0.001.

After Oil Red O staining, the plates were treated with isopropanol, and the lipid accumulation levels were measured. The absorbance values can reflect the differentiation of 3T3-L1 cells. As shown in Figure 6A, the absorbance values of the 3T3-L1 cells treated with compounds **1**, **2**, **3**, **4**, **5**, **8**, **9**, **11**, **12**, **13**, **15**, **19**, **23**, and **26** were decreased compared with the DM group to 71, 81, 63, 74, 79, 77, 75, 61, 61, 73, 81, 76, 64, and 72%, respectively in which compounds **3**, **11**, **12** and **23** were superior to compound **26** (reference compound in the positive control) in terms of reducing lipid accumulation (see Figure 6A).

Triglycerides (TGs) are the most abundant lipid component in the human body, and are also used as an index of clinical lipid-related diseases. The amount of TGs in the 3T3-L1 cells were determined. The results showed that TG levels in cells treated with compounds **1**, **2**, **3**, **4**, **9**, **11**, **12**, **13**, and **26** were decreased compared with the DM group to 72, 73, 67, 72, 64, 61, 56, 67, and 66%, respectively in which compounds **9**, **11** and **12** were greater than compound **26** (reference compound in the positive control) in terms of reducing TG accumulation and compounds **3** and **13** were very close to positive control. (see Figure 6B).

All the above results indicated that compounds **1**, **2**, **3**, **9**, **11**, **12** and **13**, especially compound **12** could inhibit the differentiation of 3T3-L1 cells and lipid accumulation. Therefore it was necessary to study the chemical constituents and their mechanisms of action in a more in-depth way.

### The Structure-Activity Relationships of the 29 Compounds

Among the 29 compounds isolated from *P. longifolia*, 12 flavonoids and nine phenolic compounds were identified. Among the 12 flavonoids, compounds **11**, **12**, and **13** exhibited significant inhibitory activity on lipid accumulation. Compared with the other nine compounds, each of these three compounds had a methoxy group at the C-3 position, rather than hydroxyl or sugar moieties. In addition, these compounds had oxygen-containing substituents at the C-4′ position, i.e. hydroxyl or methoxy groups, while there were no oxygen-containing substituents at the adjacent positions (C-3’ or C-5’). Five of the nine phenolic compounds were phenolic glycosides. According to the results of the Oil Red O staining, isopropanol decolorization, TG content, and other aspects, stronger inhibition of lipid accumulation was observed for the five phenolic glycosides compared with the non-phenolic glycosides.

### The Effects of Compound 12 on Lipid Accumulation in 3T3-L1 Cells

**FIGURE 7:**
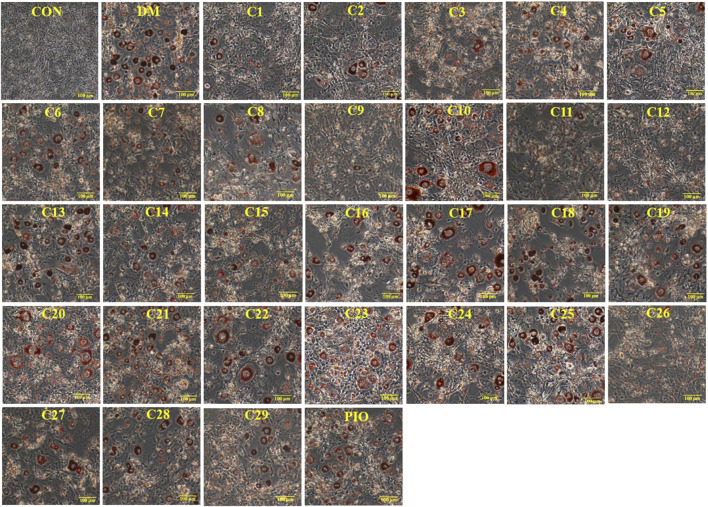
The effects of compound **12** on lipid accumulation in 3T3-L1 cells. Inhibitory effects of compound **12** on lipid accumulation were further confirmed. **(A)** Chemical structure of compound **12**. **(B)** Cell viability on 3T3-L1 cells. **(C)** The effects of compound **12** on lipid accumulation from Oil-Red O staining in 3T3-L1 cells. **(D)** Cells were treated with isopropanol and lipid accumulation was measured. **(E)** Triglyceride levels were measured by using triglyceride assay Kit. Data represent the mean ± S.D. of three separate experiments. ### *p* < 0.001 as compared with CON group. *** *p* < 0.001 compared with DM group.

From the above experimental results, compound **12** showed a considerable effect on inhibiting lipid accumulation, so we re-confirmed the cytotoxicity, photographic and quantitative assessments of lipid contents by Oil Red O staining, and TG content of compound **12** (see Figure 7), and the results were consistent with the previous experiments.

### The Effects of Compound 12 on Adipogenesis-Related Gene Expressions in 3T3-L1 Cells

To investigate the authenticity of the results of the molecular docking study, and to explore the inhibitory mechanism of compound **12** on lipid accumulation in 3T3-L1 cells, the effects of compound **12** on lipid metabolism-related genes and protein expression were studied by RT-PCR and western blot analysis.

**FIGURE 8:**
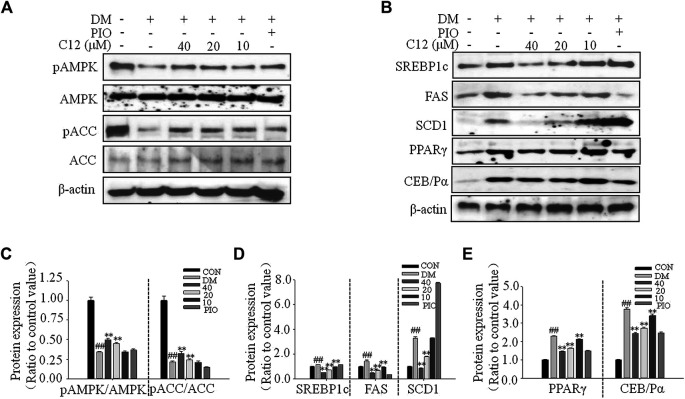
The effects of compound **12** on adipogenesis-related gene expressions in 3T3-L1 cells. **(A)** RT-PCR analysis. **(B)** Quatifications of gene expressions of ACC, SREBP1c and FAS. **(C)** Quatifications of gene expressions of SCD1 and GPAT. **(D)** Quatifications of gene expressions of PPARγ and C/EBPα. Data represent the mean ± S.D. of three separate experiments. ## *p* < 0.01 as compared with CON group. ** *p* < 0.01 compared with DM group. The significant difference was only shown to *p* < 0.01, even when the difference was *p* < 0.001.

As shown in Figure 8, compound **12** significantly inhibited the expression of genes related to adipogenesis, including SREBP1c, FAS, SCD1, GPAT, PPARγ, and C/EBPα, in a concentration-dependent manner.

### The Effects of Compound 12 on the Phosphorylations of AMPK and ACC, and Adipogenesis-Related Proteins in 3T3-L1 Cells

**FIGURE 9:**
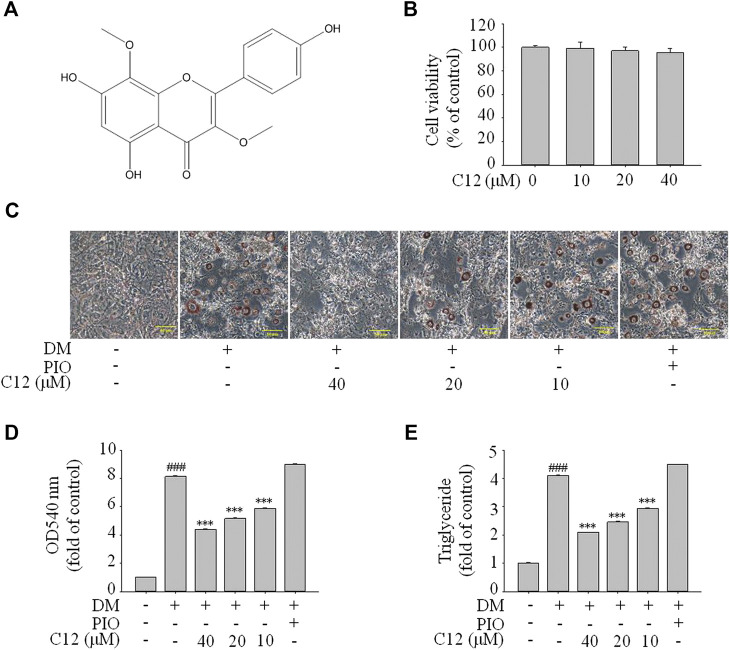
The effects of compound **12** on the phosphorylations of AMPK and ACC, and adipogenesis-related proteins in 3T3-L1 cells. **(A)** Western blot results for phosphorations of ACC and AMPK. **(B)** The effects on lipid related proteins. **(C)** Quatifications of ratios of pACC/ACC and pAMPK/AMPK. **(D)** Quatifications of protein expressions of SREBP1c, FAS, SCD1. **(E)** Quatifications of protein expressions of PPARγ and C/EBPα. Data represent the mean ± S.D. of three separate experiments. ## *p* < 0.01 as compared with CON group, ** *p* < 0.01 compared with DM group. The significant difference was only shown to *p* < 0.01, even though the difference was *p* < 0.001.

As shown in Figure 9, compound **12** significantly inhibited the protein expression of SREBP1c, FAS, SCD1, PPARγ, and C/EBPα in a concentration-dependent manner; all of these proteins are related to adipogenesis. These results were consistent with the RT-PCR results for genes related to lipid accumulation.

Compared with the DM group, the expression of pAMPK/AMPK and pACC/ACC were significantly increased, suggesting that compound **12** significantly enhanced the phosphorylations of AMPK, and inhibited the expressions of adipogenesis-related proteins and genes such as SREBP1c, FAS, and SCD1, and thereby significantly inhibited lipid accumulation.

Although there have been many studies on Potentilla species, only a few studies on chemical constituents and pharmacological activities of *P. longifolia* were conducted. In this research, the inhibitory effects of *P. longifolia* on lipid accumulation was studied for the first time. In this study, 29 compounds were isolated from 95% ethanol extract of the plant and their chemical structures were identified. It was found that three of them were new compounds and 18 compounds were isolated from this plant for the first time. Then 29 compounds were then screened for their inhibitory effects on lipid accumulation, and the structure-activity relationship was analyzed. Among the 12 flavonoids, when there was a methoxy group at the C-3 position and an oxygen-containing substituent at the C-4′ position, while there were no oxygen-containing substituents at the adjacent positions (C-3’ or C-5’), the compound seemingly showed good activity. Then, the mechanism of action of compound **12** with the best activity was studied. The results showed that adipogenesis-related proteins or genes including SREBP1c, FAS, SCD1, GPAT, PPARγ and C/EBPα, AMPK and ACC, were all more or less involved in the inhibitory effects of lipid accumulation by compound **12**.

## Conclusion

Three new and 26 known compounds were identified from the 95% ethanol extract of the dried aerial parts of *P. longifolia*. Among them, compounds **1**, **2**, **3**, **9**, **11**, **12**, and **13**, significantly inhibited the differentiation and lipid accumulation in 3T3-L1 cells. Especially, compound **12** was superior to reference compound in positive control. Western blot analysis and RT-PCR results showed that compound **12** enhanced the phosphorylation of AMPK and inhibited the expressions of adipogenesis-related proteins and genes such as SREBP1c, FAS, and SCD1, and thereby significantly inhibited lipid accumulation. *P. longifolia* and its bioactive compounds will play an important role in the treatment of diseases related to lipid accumulation in the future.

## Data Availability Statement

The raw data supporting the conclusions of this manuscript will be made available by the authors, without undue reservation, to any qualified researcher.

## Author Contributions

HY, GP, QM, LY conceived and designed the experiments; QM, LY, WL, SL, XZ performed the experiments and analyzed the data; GL, HL, CJ, YS contributed the reagents, materials, and analysis tools; CJ, HL, YS, HY, GP wrote the paper.

## Conflict of Interest

The authors declare that the research was conducted in the absence of any commercial or financial relationships that could be construed as a potential conflict of interest.

## References

[B1] Alves R. R. Rosa I. M. (2007). Biodiversity, traditional medicine and public health: where do they meet? J. Ethnobiol. Ethnomed. 3, 14 10.1186/1746-4269-3-14 17376227 PMC1847427

[B2] Arnone A. Nasini G. Merlini L. (1990). Constituents of 'Dragon's blood.' Part 4. Dracoflavan A, a novel secotriflavanoid. J. Chem. Soc., Perkin Trans. 1(22), 2637–2640. 10.1039/p19900002637

[B3] Bohr G. Gerhäuser C. Knauft J. Zapp J. Becker H. (2005). Anti-inflammatory acylphloroglucinol derivatives from hops (humuluslupulus). J. Nat. Prod. 68, 1545–1548. 10.1021/np050164z 16252923

[B4] Cha J. M. Kim D. H. Lee T. H. Subedi L. Kim S. Y. Lee K. R. (2018). Phytochemical constituents of Capsella bursa-pastoris and their Anti-inflammatory activity. Nat. Prod. Sci. 24, 132–138. 10.20307/nps.2018.24.2.132

[B5] Chu X. He X. Shi Z. Li C. Guo F. Li S. (2015). Ursolic acid increases energy expenditure through enhancing free fatty acid uptake and β-oxidation via an UCP3/AMPK-dependent pathway in skeletal muscle. Mol. Nutr. Food Res. 59, 1491–1503. 10.1002/mnfr.201400670 25944715

[B6] Gan C.-C. Ni T.-W. Yu Y. Qin N. Chen Y. Jin M.-N. (2017). Flavonoid derivative (Fla-CN) inhibited adipocyte differentiation via activating AMPK and up-regulating microRNA-27 in 3T3-L1 cells. Eur. J. Pharmacol. 797, 45–52. 10.1016/j.ejphar.2017.01.009 28088385

[B7] Gedara S. R. Abdel-Halim O. B., El-Sharkawy S. H. Salama O. M. Shier T. W. Halim A. F. (2003). New erythroxane-type diterpenoids from fagonia boveana (hadidi) hadidi & graf. Zeitschrift für Naturforschung C. 58, 23–32. 10.1515/znc-2003-1-204 12622221

[B8] Ha J.-H. Jang J. Chung S.-I. Yoon Y. (2016). AMPK and SREBP-1c mediate the anti-adipogenic effect of β-hydroxyisovalerylshikonin. Int. J. Mol. Med. 37, 816–824. 10.3892/ijmm.2016.2484 26865314

[B9] Henriksen B. S. Curtis M. E. Fillmore N. Cardon B. R. Thomson D. M. Hancock C. R. (2013). The effects of chronic AMPK activation on hepatic triglyceride accumulation and glycerol 3-phosphate acyltransferase activity with high fat feeding. Diabetol. Metab. Syndr. 5, 29 10.1186/1758-5996-5-29 23725555 PMC3679947

[B10] Hosseini-Sarvari M. Sodagar E. (2013). Esterification of free fatty acids (Biodiesel) using nano sulfated-titania as catalyst in solvent-free conditions. Compt. Rendus Chem. 16, 229–238. 10.1016/j.crci.2012.10.016

[B11] Huang S. Y. Shi J. G. Yang Y. C. Hu S. L. (2002). Study on the chemical constituents of *Coeloglossum viride* . Acta Pharm. Sin. 37, 199–203. 10.16438/j.0513-4870.2002.03.01110.1023/a:1020640114502 12579762

[B12] Ideta T. Shirakami Y. Miyazaki T. Kochi T. Sakai H. Moriwaki H. (2015). The dipeptidyl peptidase-4 inhibitor teneligliptin attenuates hepatic lipogenesis via AMPK activation in non-alcoholic fatty liver disease model mice. Int. J. Mol. Sci. 16, 29207–29218. 10.3390/ijms161226156 26670228 PMC4691103

[B13] Itoh T. Ninomiya M. Yasuda M. Koshikawa K. Deyashiki Y. Nozawa Y. (2009). Inhibitory effects of flavonoids isolated from Fragaria ananassa Duch on IgE-mediated degranulation in rat basophilic leukemia RBL-2H3. Bioorg. Med. Chem. 17, 5374–5379. 10.1016/j.bmc.2009.06.050 19596200

[B14] Jia X. H. Tang W. Z. Li J. Wang D. J. Zhang Y. Q. (2017). Chemical constituents from ethyl acetate extract of *Lonicerae Japonicae Caulis* . Chin. J. Exp. Tradit. Med. Form. 23, 62–65. 10.13422/j.cnki.syfjx.2017040062

[B15] Katashima C. K. Silva V. R. Gomes T. L. Pichard C. Pimentel G. D. (2017). Ursolic acid and mechanisms of actions on adipose and muscle tissue: a systematic review. Obes. Rev. 18, 700–711. 10.1111/obr.12523 28335087

[B16] Kurimoto S.-i. Okasaka M. Kashiwada Y. Kodzhimatov O. K. Takaishi Y. (2011). Four new glucosides from the aerial parts of *Mediasia macrophylla* . J. Nat. Med. 65, 180–185. 10.1007/s11418-010-0444-3 20640523

[B17] Li G. P. Luo Y. Li S. X. Yang C. Tian Q. Zuo M. Y. (2014). Chemical constituents in flowers of *Rhododendron lapponicum* . Chin. Tradit. Herbal. Drugs 45, 1668–1672. 10.7501/j.issn.0253-2670.2014.12.002

[B18] Li-Weber M. (2009). New therapeutic aspects of flavones: the anticancer properties of scutellaria and its main active constituents wogonin, baicalein and baicalin. Cancer Treat Rev. 35, 57–68. 10.1016/j.ctrv.2008.09.005 19004559

[B19] Lindén D. William-Olsson L. Rhedin M. Asztély A.-K. Clapham J. C. Schreyer S. (2004). Overexpression of mitochondrial GPAT in rat hepatocytes leads to decreased fatty acid oxidation and increased glycerolipid biosynthesis. J. Lipid Res. 45, 1279–1288. 10.1194/jlr.M400010-JLR200 15102885

[B20] Liu Y. Wang Z. Y. He W. J. Tan N. H. Yin Z. Q. (2015). Chemical constituents from stems and leaves of *Micromelum integerrimum* . Acta Pharm. Sin. 50, 475–479. 10.16438/j.0513-4870.2015.04.006 26223131

[B21] Liu Z. H. Yang Z. X. (2007). Isolation and identification of phytosterol from *Zantedeshia aethiopica* . China Pharm. 10, 978–979

[B22] Ma Q. Cui Y. Xu S. Zhao Y. Yuan H. Piao G. (2018). Synergistic inhibitory effects of acacetin and 11 other flavonoids isolated from *Artemisia sacrorum* on lipid accumulation in 3T3-L1 Cells. J. Agric. Food Chem. 66, 12931–12940. 10.1021/acs.jafc.8b04683 30381943

[B23] Mei Q.-X. Chen X.-L. Xia X. Fang Z.-J. Zhou H.-B. Gao Y.-Q. (2016). Isolation and chemotaxonomic significance of chemical constituents from rubus parvifolius. Chin. Herbal Med. 8, 75–79. 10.1016/S1674-6384(16)60011-4

[B24] Miao Q. Bao H. Y. Piao S. J. Lin H. Q. Qiu F. (2008). Study on chemical constituents of *Duchesnea indica* Andr. Focke. Acad. J. Second. Mil. Med. Univ. 29, 1366–1370. 10.3724/SP.J. 1008.2008.01366

[B25] Moseti D. Regassa A. Kim W.-K. (2016). Molecular regulation of adipogenesis and potential anti-adipogenic bioactive molecules. Int. J. Med. Sci. 17, 124 10.3390/ijms17010124 PMC4730365 26797605

[B26] Piao M. J. Cui S. N. Zhang D. Y. (2012). Traditional medicine of Chinese Korean nationality. Yanbian Peoples Publ. House 1–8, 194–195.

[B27] Qian S. H. Yang N. Y. Duan J. A. Yuan L. H. Tian L. J. (2004). Study on the flavonoids of *Eupatorium lindleyanum* . China J. Chin. Mater. Med. 29, 69–71. 15709382

[B28] Rabesa Z. A. Voirin B. (1978). Un nouvel aglycone C-methyl flavonique, le C-methyl-6 0-methyl-3 kaempferol isole de. Tetrahedron Lett. 19, 3717–3718. 10.1016/s0040-4039(01)95039-7

[B29] Shu W. H. Zhang Y. Ye W. C. Zhou G. X. (2011). Glycoside constituents from solanum torvum swartz. J. Jinan. Univ. Natur. Sci. 32, 493–497.

[B30] Sut S. Dall’Acqua S. Uysal S. Zengin G. Aktumsek A. Picot-Allain C. (2019). LC-MS, NMR fingerprint of *Potentilla argentea* and *Potentilla recta* extracts and their *in vitro* biopharmaceutical assessment. Ind. Crop. Prod. 131, 125–133. 10.1016/j.indcrop.2019.01.047

[B31] Tomczyk M. Latté K. P. (2009). Potentilla-A review of its phytochemical and pharmacological profile. J. Ethnopharmacol. 122, 184–204. 10.1016/j.jep.2008.12.022 19162156

[B32] Uysal S. Zengin G. Locatelli M. Bahadori M. B. Mocan A. Bellagamba G. (2017). Cytotoxic and enzyme inhibitory potential of two Potentilla species (*P. speciosa* L. and *P. reptans* Willd.) and their chemical composition. Front. Pharmacol. 8, 112–118. 10.3389/fphar.2017.00290 28588492 PMC5441381

[B33] Wada K. Sakamoto H. Nishikawa K. Sakuma S. Nakajima A. Fujimoto Y. (2007). Life style-related diseases of the digestive system: endocrine disruptors stimulate lipid accumulation in target cells related to metabolic syndrome. J. Pharmacol. Sci. 105, 133–137. 10.1254/jphs.FM0070034 17928741

[B34] Wang A. P. Liu M. C. Yang S. J. Hu D. Y. Yang S. (2011). Chemical constituents of *Cudrania tricuspidata* . Chin. J. Exp. Tradit. Med. Form. 17, 113–115. 10.13422/j.cnki.syfjx.2011.15.046

[B35] Wang D. Lao L. Pang X. Qiao Q. Pang L. Feng Z. (2018). Asiatic acid from Potentilla chinensis alleviates non-alcoholic fatty liver by regulating endoplasmic reticulum stress and lipid metabolism. Int. Immunopharm. 65, 256–267. 10.1016/j.intimp.2018.10.013 30340105

[B36] Wang W. Zeng Y. H. Osman K. Shinde K. Rahman M. Gibbons S. (2010). Norlignans, acylphloroglucinols, and a dimeric xanthone from Hypericum chinense. J. Nat. Prod. 73, 1815–1820. 10.1021/np1004483 21043475

[B37] Wang Y.-D. Bao X.-Q. Xu S. Yu W.-W. Cao S.-N. Hu J.-P. (2016). A novel Parkinson's disease drug candidate with potent anti-neuroinflammatory effects through the Src signaling pathway. J. Med. Chem. 59, 9062–9079. 10.1021/acs.jmedchem.6b00976 27617803

[B38] Wang Z. He Y. C. Wang D. Cheng R. R. Yang C. R. Xu M. (2014a). Safety analysis for aircraft one engine inoperation during departure. Amministrare 614, 631–634. 10.5012/bkcs.2014.35.2.631

[B39] Wang Z. W. Xu X. H. Chen X. T. Yu S. S. Liu H. D. Hayashi T. (2014b). Chemical constituents from the aerial part of *Sibiraea angustata* . Chin. Med. Mat. 37, 57–60. 10.1016/j.phytol.2013.05.016 25090704

[B40] Wasowski C. Marder M. Viola H. Medina J. H. Paladini A. C. (2002). Isolation and identification of 6-methylapigenin, a competitive ligand for the brain GABAAReceptors, from*Valeriana wallichii* . Planta Med. 68, 934–936. 10.1055/s-2002-34936 12391561

[B41] Wollenweber E. Dörr M. (2008). Flavonoid aglycones from the lipophilic exudates of some species of Rosaceae. Biochem. Systemat. Ecol. 36, 481–483. 10.1016/j.bse.2007.12.004

[B42] Wu J. Mu R. Sun M. Zhao N. Pan M. Li H. (2019). Derivatives of natural product agrimophol as disruptors of intrabacterial pH homeostasis in *Mycobacterium tuberculosis* , ACS Infect. Dis. 5, 1087–1104. 10.1021/acsinfecdis.8b00325 31016962

[B43] Yang B.-Y. Chen Z.-L. Liu Y. Guo J.-T. Kuang H.-X. (2018). New lignan from the rattan stems of *Schisandra chinensis* . Nat. Prod. Res. 33, 340–346. 10.1080/14786419.2018.1452000 29544361

[B44] Yang B. Y. Xia Y. G. Chen D. Kuang H. X. (2010). Chemical constituents from the flower of datura metel. Chin. J. Nat. Med. 8, 429–432. 10.1016/j.foodchem.2020.127714

[B45] Yang D. Wang L. Zhai J. X. Han Na. Liu Z. H. Li S. K. (2020). Characterization of antioxidant, α-glucosidase and tyrosinase inhibitors from the rhizomes of *Potentilla anserina* L. and their structure-activity relationship. Food Chem. 336, 127714 10.1016/j.foodchem.2020.127714 32828014

[B46] Yuan H. Ma Q. Ye L. Piao G. (2016). The traditional medicine and modern medicine from natural products. Molecules 21, 559 10.3390/molecules21050559 PMC6273146 27136524

[B47] Zhou Y. Y. Wang D. Guan F. (2007). Studies on the active compounds to relieve cough and dyspnea from *Rhododendron dauricum* Lishizhen. Med. Mater. Med. Res. 18, 2461–2462.

