# Supplementary material for: Inhibitory Effects of Twenty-Nine Compounds From Potentilla longifolia on Lipid Accumulation and Their Mechanisms in 3T3-L1 Cells
Source: Front Pharmacol. 2020 Nov 9;11:555715. doi: 10.3389/fphar.2020.555715 (PMC7680851; doi:10.3389/fphar.2020.555715)
Supplement: Supplementary file 1 [file DataSheet1_v1.PDF]

Supplementary data

Title: Inhibitory Effects of Thirty Compounds from *Potentilla longifolia* on Lipid Accumulation and Their Mechanisms in 3T3-L1 Cells

Qianqian Ma[a,#], Li Ye[a,#], Wei Li[a], Shengxi Lin[a], Xiaoyan Zhao[a], Chenghua Jin[a], Guancheng Liu[a], Huan Liu[a], Yunpeng Sun[a], Haidan Yuan[a,b,*], Guangchun Piao [a,b,*]

[a] *College of Pharmacy, Yanbian University; Yanji, Jilin, 133002, China.*

[b] *Key Laboratory of Natural Resources of Changbai Mountain & Functional Molecules (Yanbian University), Ministry of Education, China.*

*Corresponding authors: **Haidan Yuan**, College of Pharmacy, Yanbian University; 977 Gongyuan Street, Yanji 133002, China. E-mail: hdyuan@ybu.edu.cn; **Guangchun Piao**, College of Pharmacy, Yanbian University; 977 Gongyuan Street, Yanji 133002, China. E-mail: gcpiao@ybu.edu.cn.

[#] These authors contributed equally to the work.

ABSTRACT: *Potentilla longifolia* Willd. ex Schlecht, which is a kind of traditional Chinese herb, is often referred to as “Ganyancao” in China, which means “the herb is effective in the treatment of liver inflammation”. However, there are little modern studies on its chemical constituents and their pharmacological effects so far. Three new (ganyearmcaoosides A and B and ganyearmcaoic acid A; 1–3) and 27 known compounds (4–30) were isolated from the 95% ethanol extract of the dried aerial parts of this plant, of which 23 were isolated for the first time from this plant. The chemical structures of these compounds were elucidated using 1D and 2D NMR (^{1}H−^{1}H COSY, HMBC, etc.) and HR-ESI-MS analysis. Cytotoxicity assessment was carried out in 3T3-L1 cells exposed to various concentrations (10-80 mM) of 30 compounds for 96 h using MTT assay. The inhibitory effects of the 30 compounds with safe concentrations on the lipid accumulation in 3T3-L1 cells were evaluated using photographic and quantitative assessments of lipid contents by Oil Red O staining, and measurement of the triglyceride levels. Comprehensive analysis from several aspects of these screening experiments showed that compounds 1, 2, 3, 9, 11, 12, and 13 significantly inhibited the differentiation and lipid accumulation in 3T3-L1 cells. Among them, compound 12 (3,8-dimethoxy-5,7,4'- trihydroxyflavone) showed the best inhibitory effect on lipid accumulation such as reducing the accumulation of oil droplets and triglyceride level, and was superior to the reference (ursolic acid) in positive control. Molecular docking analysis showed that compound 12 had greater binding affinities with AMPK than the reference compound. Western blot analysis and RT-PCR results showed that compound 12 enhanced the phosphorylations of AMPK and ACC, and inhibited the expressions of adipogenesis-related proteins and genes including SREBP1c, FAS, SCD1, GPAT, PPARγ and C/EBPα, and thereby

significantly inhibited lipid accumulationin in a concentration-dependent manner. The results from this study revealed that *P. Longifolia* and its bioactive compounds could be promising as potential therapeutic agents for diseases related to lipid accumulation in the future.

Keywords: *Potentilla longifolia*, lipid accumulation, 3,8-dimethoxy-5,7,4'-trihydroxyflavone, SREBP1c, PPARγ, molecular docking, new compound (ganyearmcaooside)

LIST of FIGURES

FIGURE S1. ^{1}H NMR spectrum of compound 1 in CD_3OD (300MHz).

FIGURE S2.^{13}C NMR spectrum of compound 1 in CD_3OD (75MHz).

FIGURE S3. HMQC spectrum of compound 1 in CD_3OD.

FIGURE S4. HMBC spectrum of compound 1 in CD_3OD.

FIGURE S5. HR-ESI-MS spectrum of compound 1.

FIGURE S6. ^{1}H NMR spectrum of compound 2 in DMSO (500MHz).

FIGURE S7.^{13}C NMR spectrum of compound 2 in DMSO (125MHz).

FIGURE S8. HMQC spectrum of compound 2 in DMSO.

FIGURE S9. HMBC spectrum of compound 2 in DMSO.

FIGURE S10. HR-ESI-MS spectrum of compound 2.

FIGURE S11. ^{1}H NMR spectrum of compound 3 in CD_3OD (300MHz).

FIGURE S12.^{13}C NMR spectrum of compound 3 in CD_3OD (75MHz).

FIGURE S13. ^{1}H–^{1}H COSY spectrum of compound 3 in CD_3OD.

FIGURE S14. NOESY spectrum of compound 3 in CD_3OD.

FIGURE S15. HMQC spectrum of compound 3 in CD_3OD.

FIGURE S16. HMBC spectrum of compound 3 in CD_3OD.

FIGURE S17. HR-ESI-MS spectrum of compound 3.

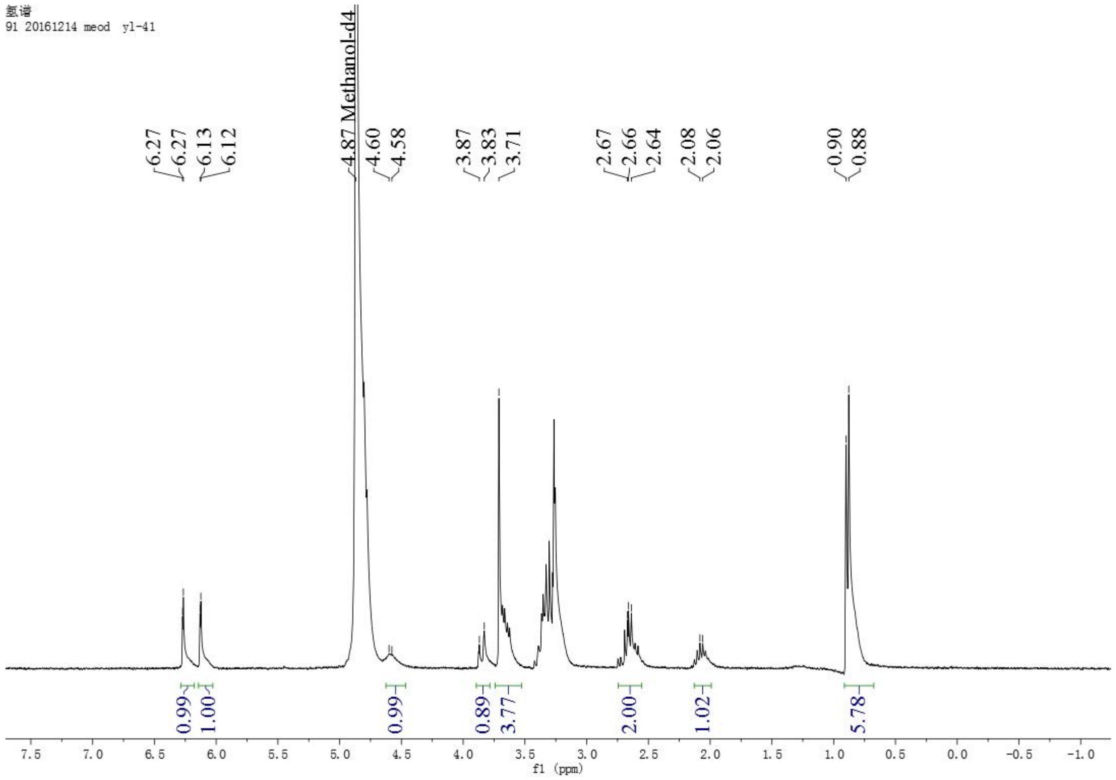

FIGURE S1. ^{1}H NMR spectrum of compound 1 in CD_3OD.

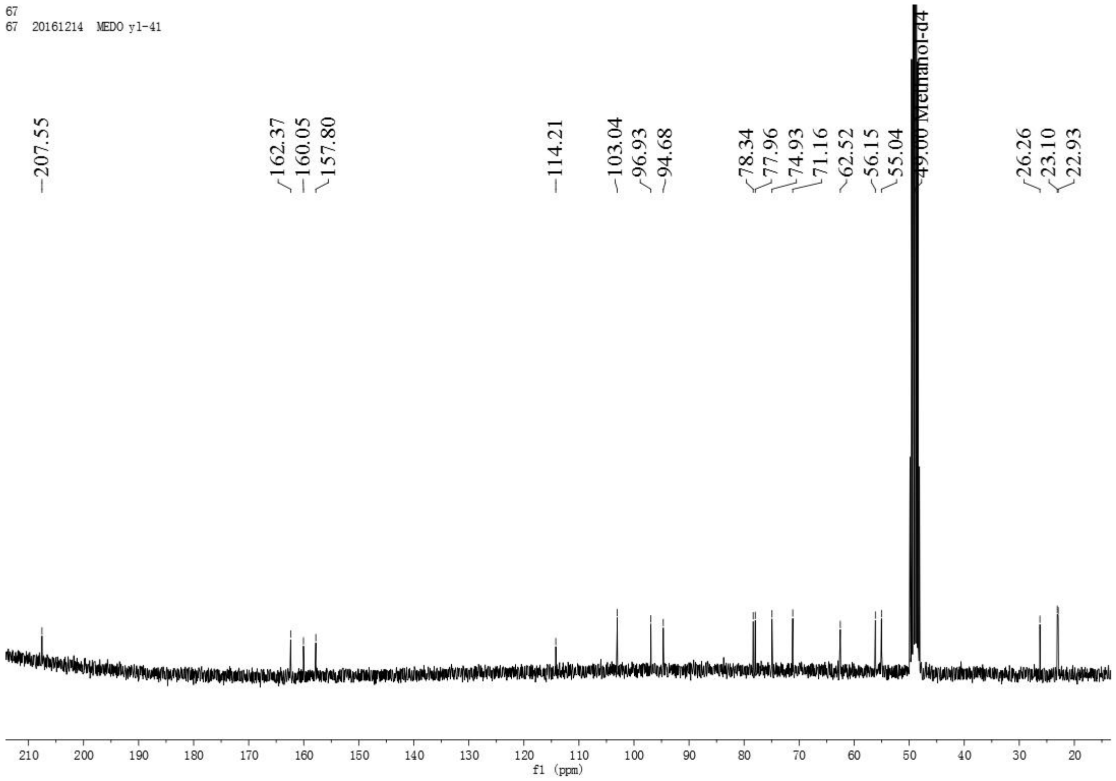

FIGURE S2. ^{13}C NMR spectrum of compound 1 in CD_3OD.

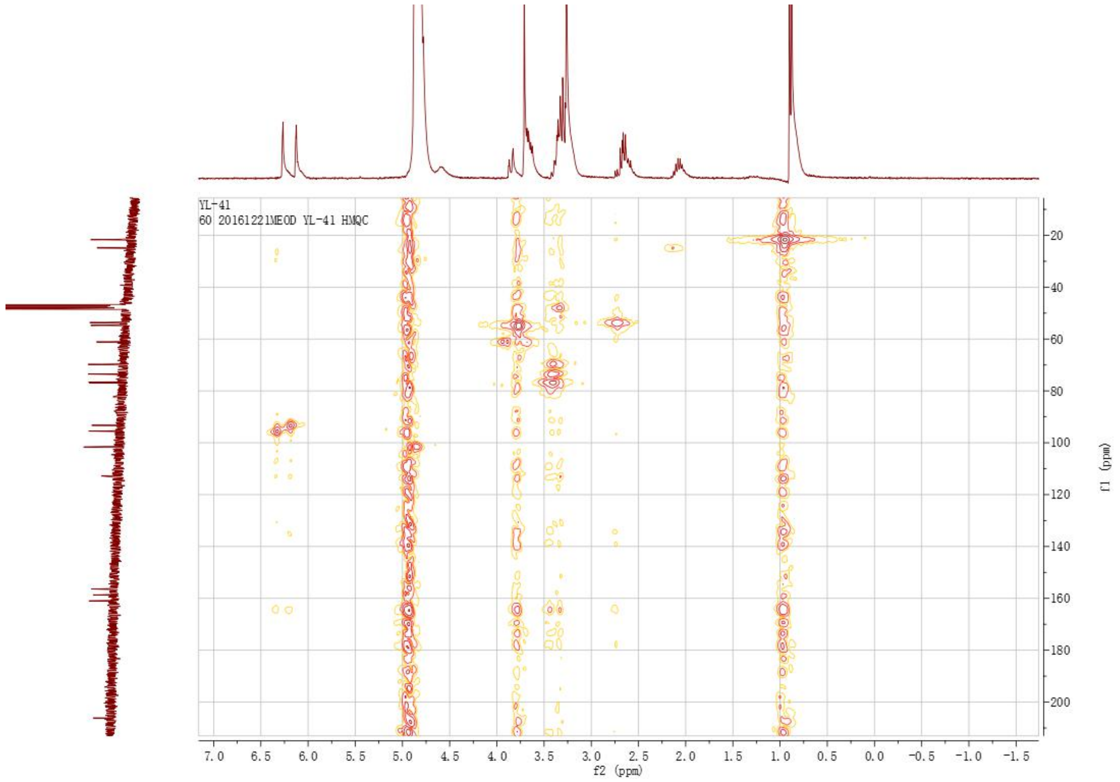

FIGURE S3. HMQC spectrum of compounds 1 in CD_3OD.

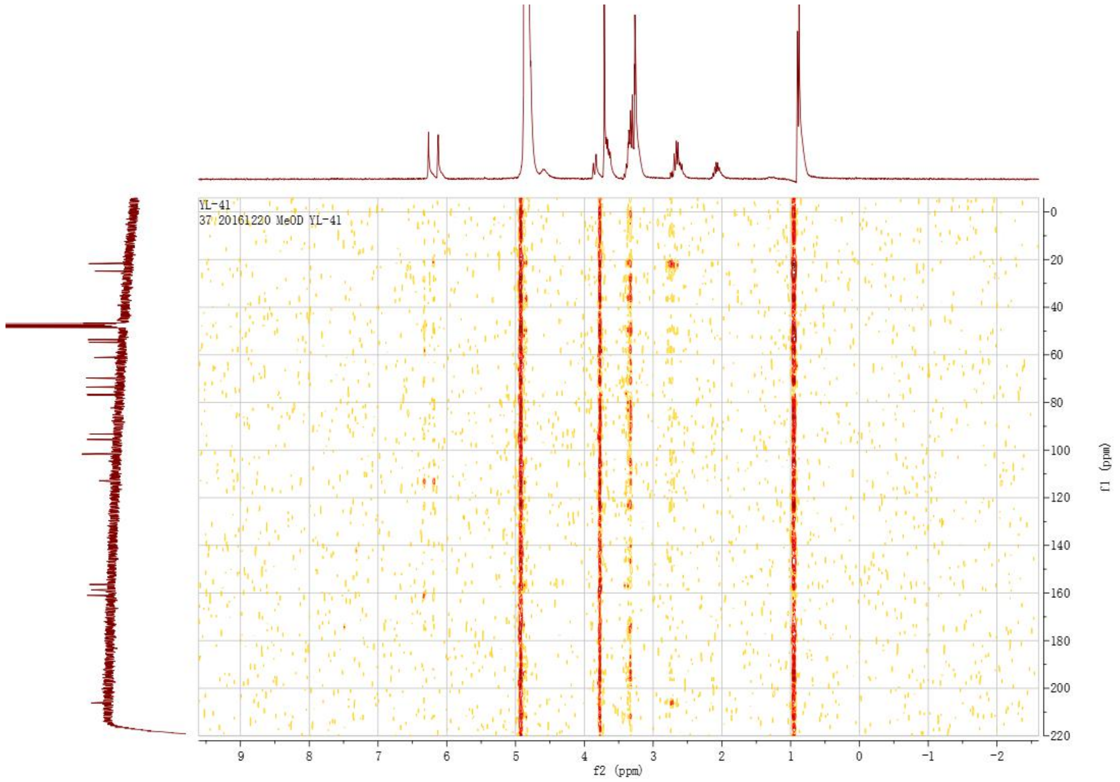

FIGURE S4. HMBC spectrum of compound 1 in CD_3OD.

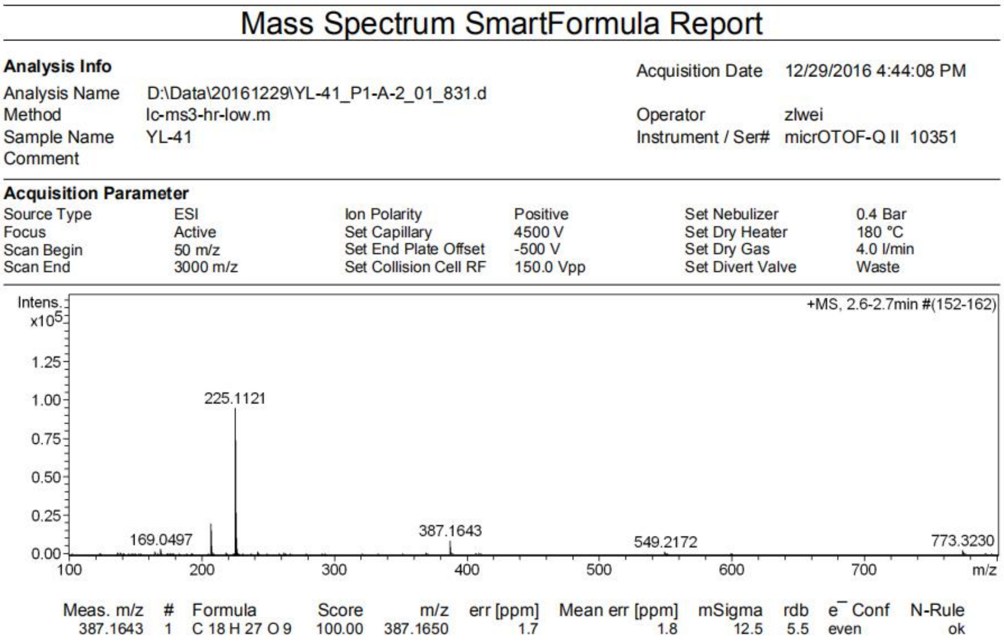

FIGURE S5. HR-ESI-MS spectrum of compound 1.

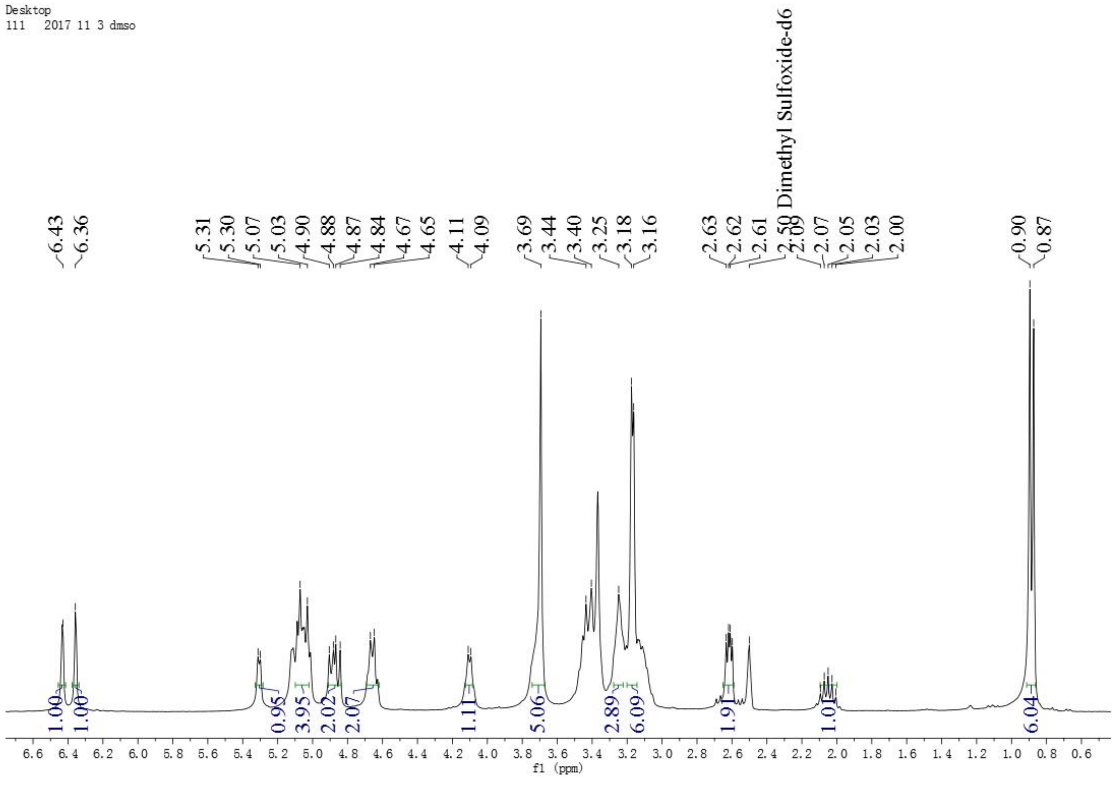

FIGURE S6. ^{1}H NMR spectrum of compound 2 in DMSO.

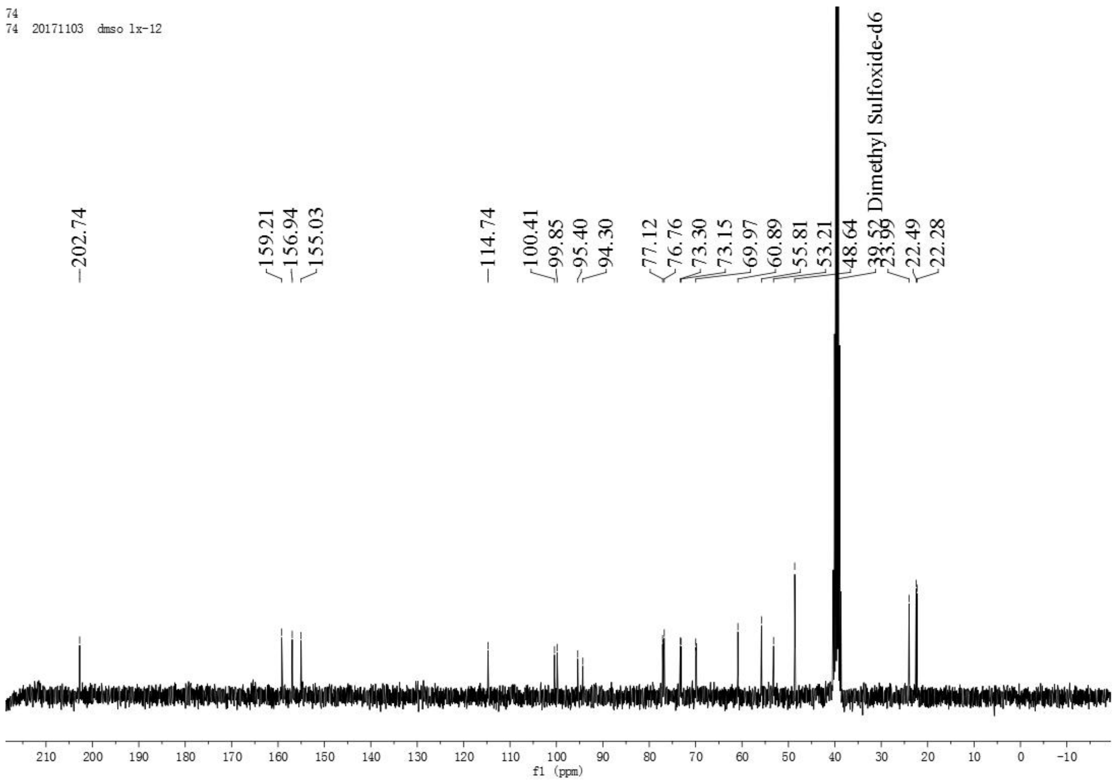

FIGURE S7.^{13}C NMR spectrum of compound 2 in DMSO.

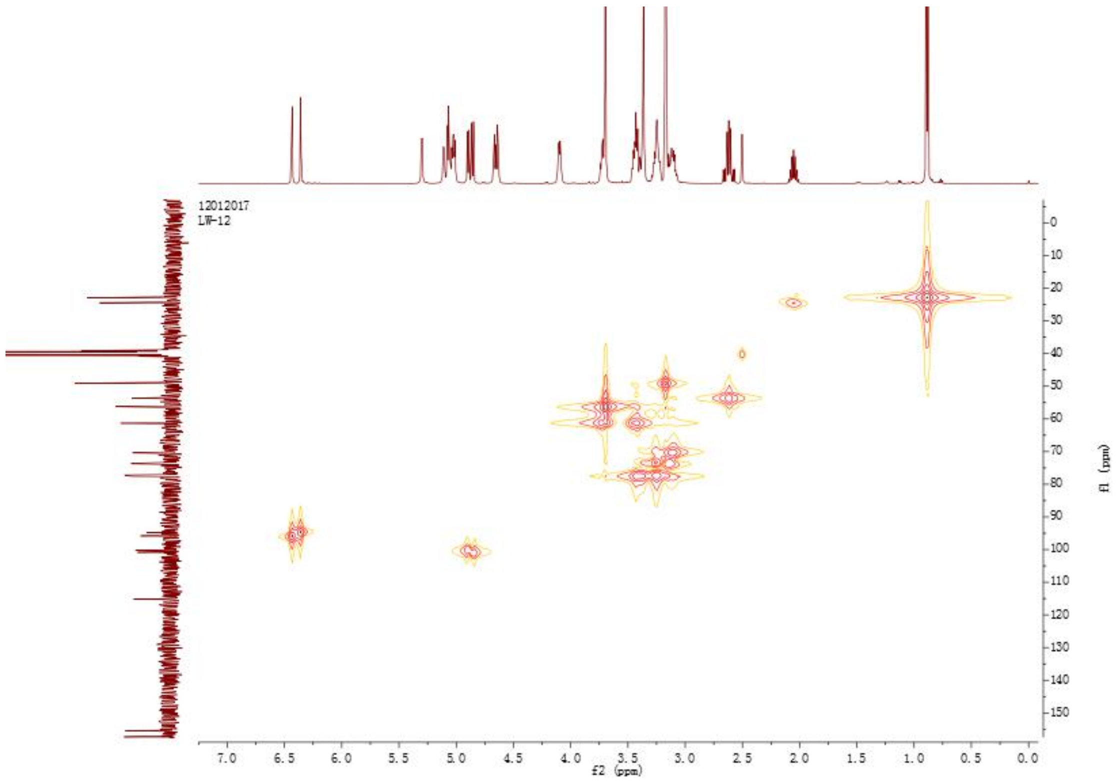

FIGURE S8. HMQC spectrum of compound 2 in DMSO.

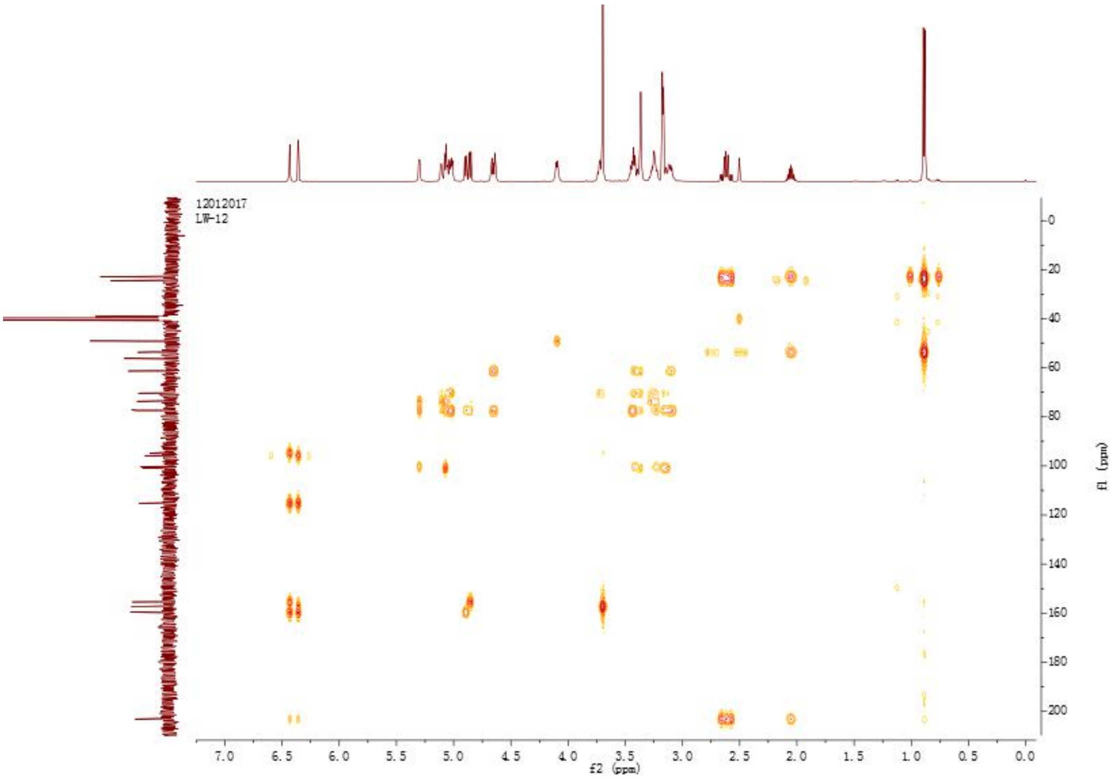

FIGURE S9. HMBC spectrum of compound 2 in DMSO.

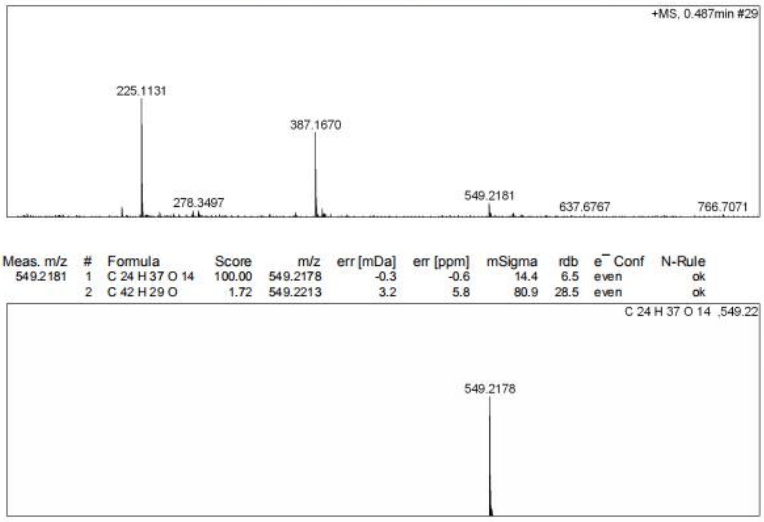

Mass Spectrum SmartFormula Report

Analysis Info

Analysis Name	D:\Data\20180116\lw-12_P1-B-1_01_5024.d	Acquisition Date	1/16/2018 1:07:13 PM
Method	lc-ms-hr-low.m	Operator	zlwei
Sample Name	lw-12	Instrument / Ser#	micrOTOF-Q II 10351
Comment			

Acquisition Parameter

Source Type	ESI	Ion Polarity	Positive	Set Nebulizer	0.4 Bar
Focus	Active	Set Capillary	4500 V	Set Dry Heater	200 °C
Scan Begin	50 m/z	Set End Plate Offset	-500 V	Set Dry Gas	5.0 l/min
Scan End	1200 m/z	Set Collision Cell RF	100.0 Vpp	Set Divert Valve	Waste

Meas. m/z	#	Formula	Score	m/z	err [mDa]	err [ppm]	mSigma	rdb	e⁻ Conf	N-Rule
549.2181	1	C 24 H 37 O 14	100.00	549.2178	-0.3	-0.6	14.4	6.5	even	ok
	2	C 42 H 29 O	1.72	549.2213	3.2	5.8	80.9	28.5	even	ok

Meas. m/z	#	Formula	Score	m/z	err [mDa]	err [ppm]	mSigma	rdb	e⁻ Conf	N-Rule

FIGURE S10. HR-ESI-MS spectrum of compound 2.

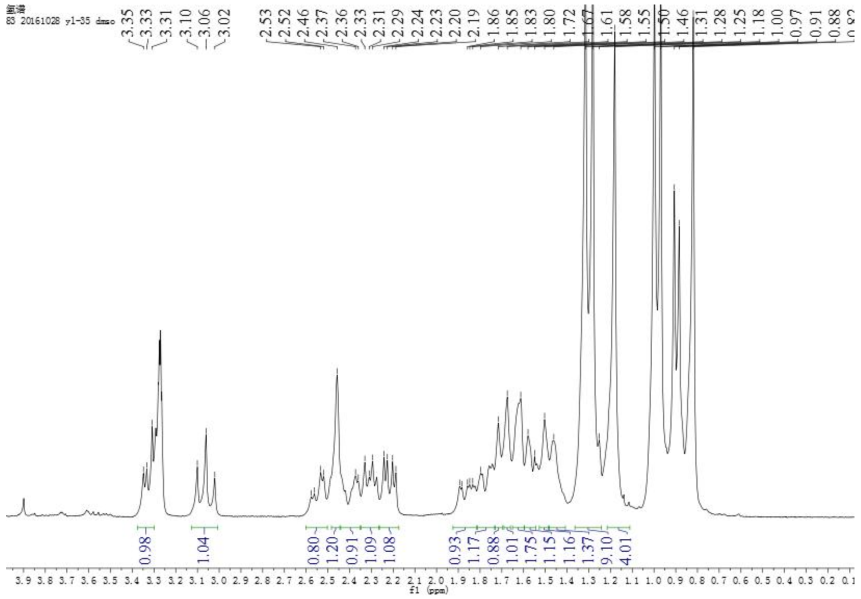

FIGURE S11.^{1}H NMR spectrum of compound 3 in CD_3OD.

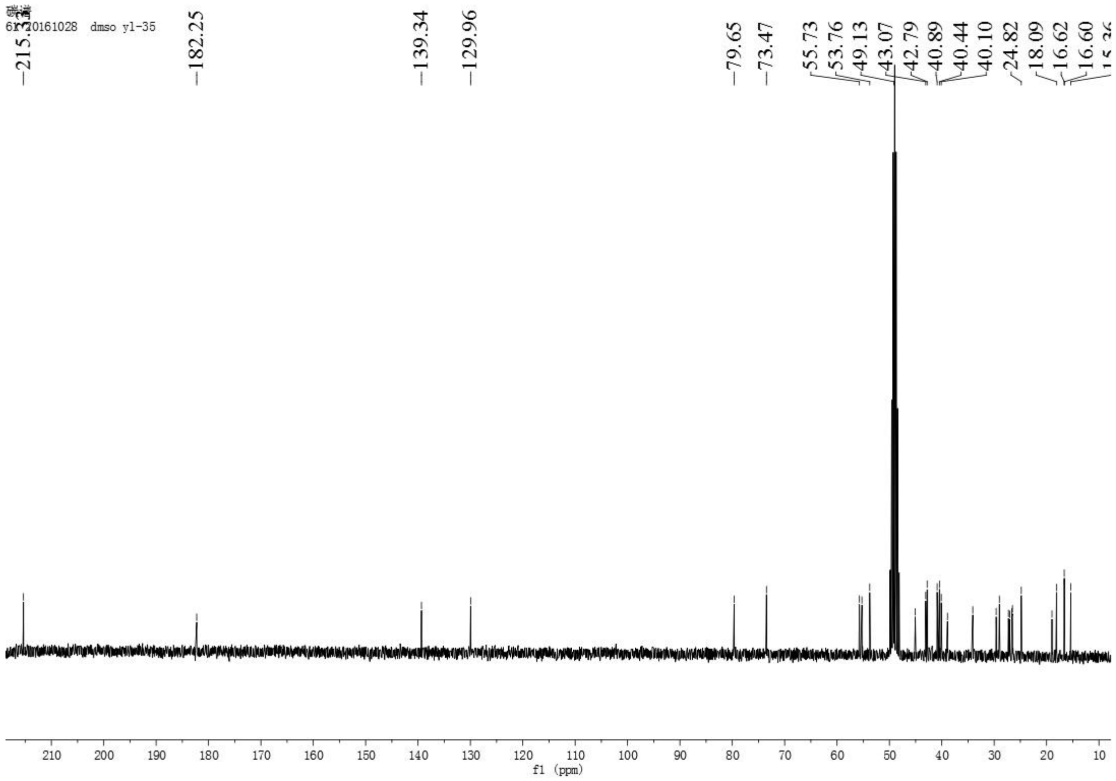

FIGURE S12.^{13}C NMR spectrum of compound 3 in CD_3OD.

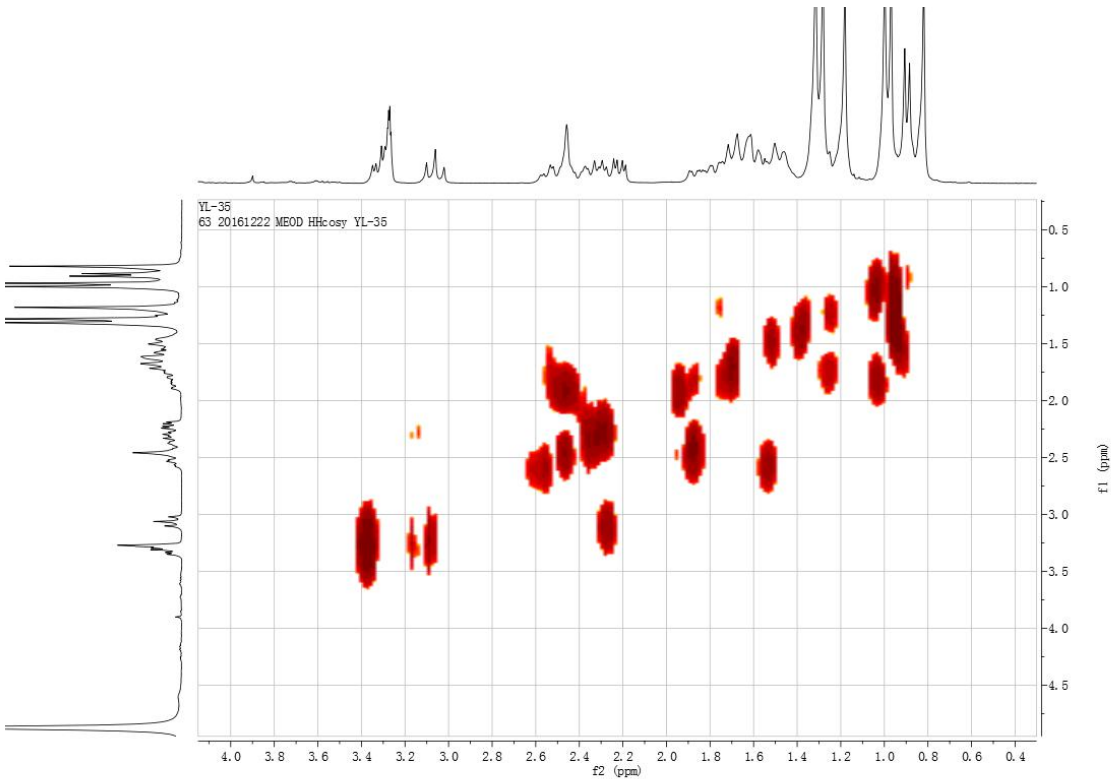

FIGURE S13. $^{1}H-^{1}H$ COSY spectrum of compound 3 in CD_3OD.

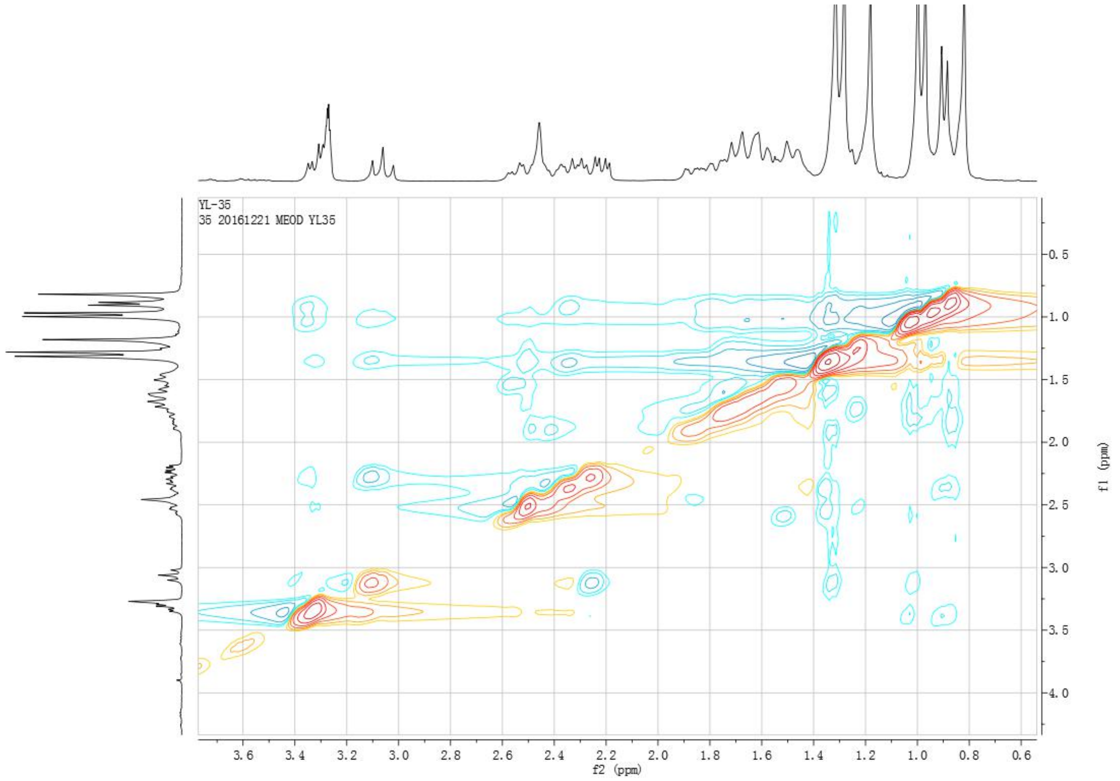

FIGURE S14. NOESY spectrum of compound 3 in CD_3OD.

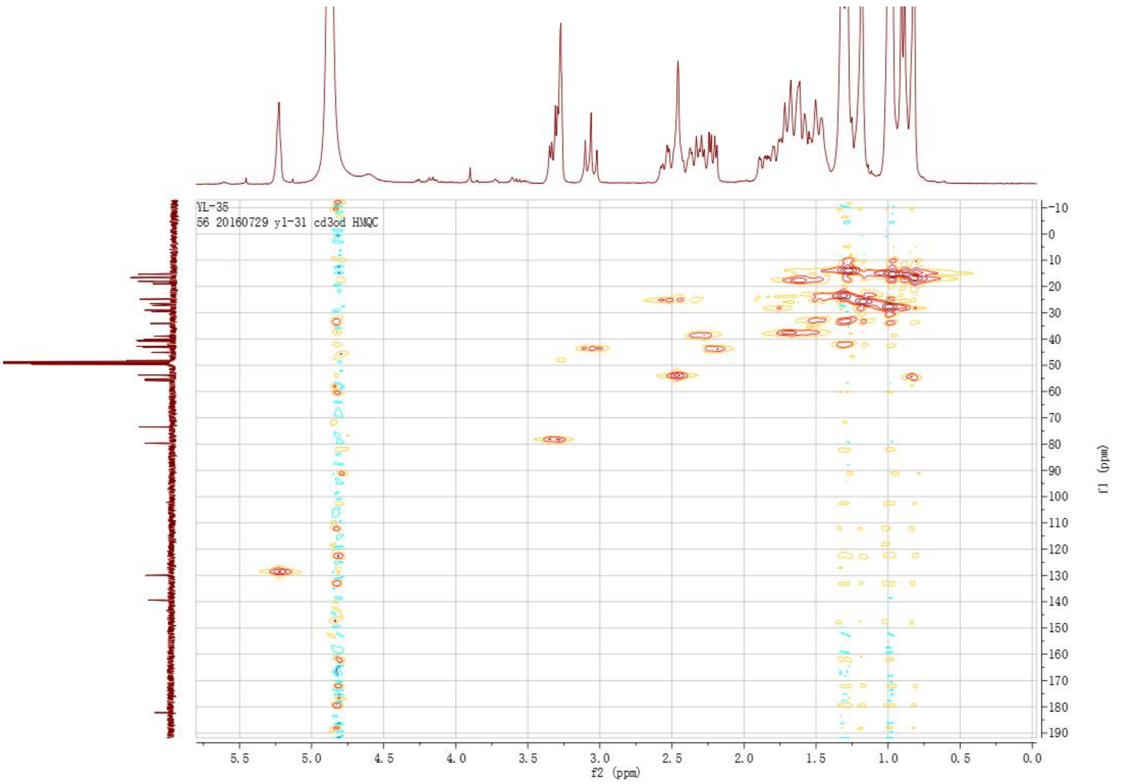

FIGURE S15. HMQC spectrum of compound 3 in CD_3OD.

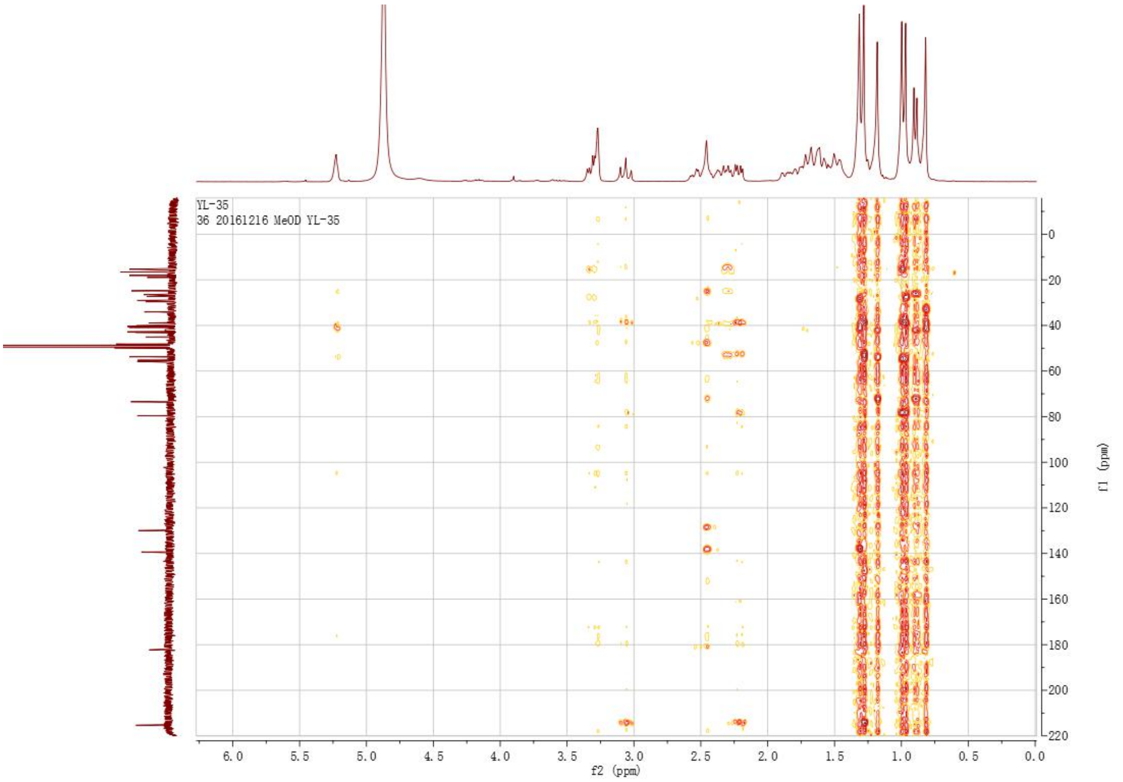

FIGURE S16. HMBC spectrum of compound 3 in CD_3OD.

Mass Spectrum SmartFormula Report

Analysis Info

Analysis Name	D:\Data\20161229\YL-35_P1-A-1_01_830.d	Acquisition Date	12/29/2016 4:37:06 PM
Method	lc-ms3-hr-low.m	Operator	zlwei
Sample Name	YL-35	Instrument / Ser#	micrOTOF-Q II 10351
Comment			

Acquisition Parameter

Source Type	ESI	Ion Polarity	Positive	Set Nebulizer	0.4 Bar
Focus	Active	Set Capillary	4500 V	Set Dry Heater	180 °C
Scan Begin	50 m/z	Set End Plate Offset	-500 V	Set Dry Gas	4.0 l/min
Scan End	3000 m/z	Set Collision Cell RF	150.0 Vpp	Set Divert Valve	Waste

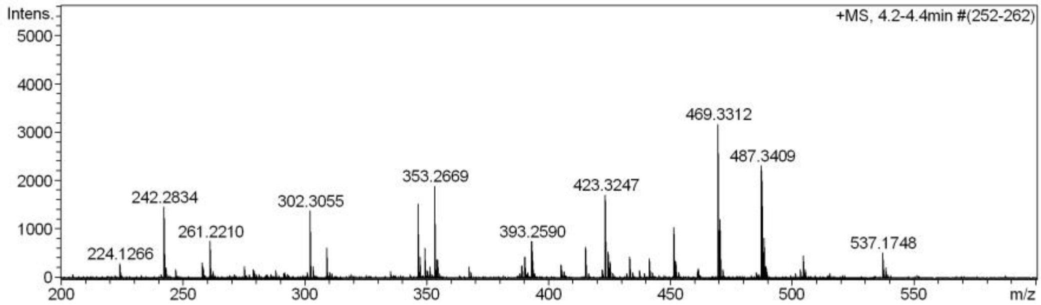

Meas. m/z	#	Formula	Score	m/z	err [ppm]	Mean err [ppm]	mSigma	rdb	e⁻ Conf	N-Rule
487.3409	1	C 30 H 47 O 5	100.00	487.3418	1.8	2.5	24.6	7.5	even	ok

FIGURE S17. HR-ESI-MS spectrum of compound 3.
